# IV Vitamin C in Sepsis: A Latest Systematic Review and Meta-Analysis

**DOI:** 10.1155/2023/6733465

**Published:** 2023-01-24

**Authors:** Chengli Wen, Yuan Li, Qinxue Hu, Hui Liu, Xinxin Xu, Muhan Lü

**Affiliations:** Affiliated Hospital of Southwest Medical University, 25 Taiping Street, Jiangyang District, Luzhou, Sichuan, China

## Abstract

Sepsis is a high-incidence disease and demands intensive care. Finding effective treatment is the key to cure sepsis. Studies have shown a lower level of vitamin C in patients with sepsis. Therefore, vitamin C supplementation has become one of the measures to treat sepsis. However, the clinical studies of vitamin C in the treatment of sepsis have been controversial. We performed a meta-analysis to evaluate vitamin C's efficacy and safety in the treatment of sepsis. We searched four electronic databases: PubMed, Embase, Web of Science, and the Cochrane Library, and two researchers independently screened 24 eligible RCTs published in English. Our review demonstrates that intravenous (IV) vitamin C might improve short-term mortality (RR, 0.82; 95% CI, 0.65–1.02; *P*=0.07; and *I*^2^ = 45%) and overall mortality (RR, 0.86; 95% CI, 0.74–1.01; *P*=0.06; and *I*^2^ = 51%) of patients with sepsis. Moreover, the SOFA score of patients with sepsis improved significantly after treatment with vitamin C for over 72 hours (RR, 0.26; 95% CI, 0.09–0.42; *P*=0.002; and *I*^2^ = 0%). The main results of our study were moderate-quality evidence. More high-quality, multicenter RCTs are needed to provide more substantial evidence on the efficacy and safety of IV vitamin C for sepsis.

## 1. Introduction

Sepsis is a life-threatening condition caused by a dysregulated host response to infection and could lead to organ dysfunction [1]. Sepsis is a common disease in the intensive care unit, which is the leading cause of death from infection and major healthcare problems. The occurrence of sepsis was close to 50 million cases, while about 20% of all-cause deaths worldwide were sepsis-related [2]. A meta-analysis estimated the hospital mortality for sepsis to be close to 27% [3]. The mortality for sepsis can be as high as 46.7%, which is much higher than that in other critically ill patients [2]. Despite differences in medical expenditure between countries and regions [4], sepsis results in high healthcare costs due to its high incidence and the need for high-level medical care for critically ill individuals. The total medical expenditures related to sepsis are the highest in the United States (€51,671 × 10^6^) [5]. Early diagnosis and effective treatment of sepsis can reduce patients with severe sepsis, thereby reducing medical costs [6].

Vitamin C, also called ascorbic acid, can impact the expression of coagulation and proinflammatory genes. It can also regulate the immune system, maintain circulating cytokine homeostasis, and exhibit anti-inflammatory and antioxidant properties [7]. The level of vitamin C is often lower in patients with sepsis [8, 9]; therefore, vitamin C supplementation has become one of the measures to treat sepsis. Clinical studies of vitamin C in the treatment of sepsis have shown mixed results on the mortality of patients. Some studies suggest that vitamin C could significantly reduce mortality in patients with sepsis [10–12], while others did not show any effect [13, 14]. The use of vitamin C in the treatment of sepsis is controversial. There have been many high-quality randomized controlled studies on the treatment of sepsis with vitamin C in the last two years [13, 15–17]. We attempt to evaluate the efficacy and safety of vitamin C supplementation in patients with sepsis through a systematic review and meta-analysis of randomized controlled trials (RCTs).

## 2. Methods

This systematic review and meta-analysis were conducted based on the Preferred Reporting Items for Systematic Reviews and Meta-Analyses (PRISMA), 2020 [18].

The research protocol has been registered on the international prospective register of systematic reviews (PROSPERO), with CRD42022350810 as the registration number.

### 2.1. Data Sources and Search Strategy

Four electronic databases, including PubMed, Embase, Web of Science, and the Cochrane Library, were searched independently by two researchers (Chengli Wen and Qinxue Hu) to explore eligible literature published in English from the establishment of the databases until August 2022. The databases were searched by medical subject headings and keywords, which included “vitamin C,” “ascorbic acid,” “sepsis,” and “septic shock.”

### 2.2. Eligibility Criteria

Inclusion criteria for the study were as follows: (1) study design: RCT; (2) participants: adult patients with sepsis or septic shock; (3) intervention: vitamin C administered intravenously (IV), given alone or in combination with thiamine and/or corticosteroids; and (4) result: mortality or length of hospital/ICU stay. Exclusion criteria were as follows: (1) study designs other than RCTs; (2) studies involving animals or minors; (3) non-IV mode of vitamin C administration; and (4) outcomes without mortality or length of hospital/ICU stay.

### 2.3. Data Extraction

The literature was screened and extracted based on the eligibility criteria independently by two researchers (Chengli Wen and Qinxue Hu). In case of disagreements, the data were discussed with a third researcher (Hui Liu). The information obtained from the eligible studies included the first author of the studies, the publication year, the country, study design, sample size, intervention details, mortality (28-day, 30-day, 90-day, 180-day, ICU, hospital, and all-cause mortalities), duration of vasopressors, ∆Sequential Organ Failure Assessment (SOFA) (72 h), new onset of acute kidney injury (AKI), duration of mechanical ventilation, adverse events, length of ICU, and hospital stay.

### 2.4. Study Quality Assessment

The quality of the studies was independently assessed by two researchers (Chengli Wen and Qinxue Hu) based on the Cochrane Collaboration's tool and modified Jadad scale. The disagreements between the two authors were resolved in consultation with the third researcher (Hui Liu). Cochrane is a tool for measuring risk assessment that assigns a high-risk, low-risk, or unclear-risk assessment on key points, including the generation of random sequence, the allocation concealment method, participants' blinding, data collectors, healthcare professionals, outcome assessors, and data analysts, and incomplete outcome data [19]. The modified Jadad scale score was determined from four aspects: (a) randomization (0: not randomized or the randomization method was inappropriate, 1: the study was described as randomized, and 2: the study used a suitable randomization method), (b) allocation concealment (0: the study did not explain the method of concealed allocation, 1: the study illustrated the allocation concealment method, and 2: the technique of concealed allocation was explained adequately), (c) double blinding (0: no blinding or improper blinding technique, 1: the study employed double-blind technique, and 2: the appropriate double blinding technique was described), and (d) withdrawals and dropouts (0: the study did not describe the follow-ups and 1: the study described the withdrawals and dropouts). The modified Jadad scale scores of 1–3 and 4–7 were considered low-quality and high-quality studies, respectively [20].

### 2.5. Outcome Measures

The primary outcome was the overall mortality, defined as mortality at the end of the follow-up for each study. Different studies followed up for different periods; therefore, the overall mortality could be one of the 28-day, 30-day, 90-day, 180-day, hospital, ICU, and all-cause mortalities in literature. The secondary outcomes included 28-day, 30-day, 90-day, 180-day, ICU, and hospital mortalities, as well as new onset of AKI, adverse events, duration of vasopressors, ∆SOFA (72 h), and duration of mechanical ventilation.

### 2.6. Effect Measures

Dichotomous results were reported using relative hazard ratios (RR), while continuous results were reported using standardized uniform differences (SMD), as the units of the results were different.

### 2.7. Synthesis Methods for Data

For data processing, the method reported by Wan et al. [21]and Luo et al. [22] was used; the median was converted into the mean depending on the study's sample size. When the sample size was greater than 50, the method provided by Luo et al. was chosen; otherwise, the method by Wan et al. was employed. Similar interventions were compared using the random effect meta-analysis. RevMan 5.3 (Cochrane IMS, Oxford, United Kingdom) was used for all analyses with a random-effect model. Stata 14.0 MP-Parallel Edition (College Station, TX, USA) was used to do the funnel plot, Egger's test, and meta-regression. Heterogeneity was measured using the chi-squared test and *I*^2^ statistics. The value *I*^2^ greater than 50% and *P* less than 0.05 indicated statistical heterogeneity. When heterogeneity was evident and the number of literature was more than 20, meta-regression, subgroup, and sensitivity analyses were used to identify the sources of the heterogeneity. Furthermore, Egger's test and the funnel plot were used to detect publication bias when the number of literature was greater than or equal to 10. All estimates were reported with a 95% confidence interval (CI).

### 2.8. Subgroup Analyses

Subgroup analyses were performed to find the sources of heterogeneity in the clinical outcomes. The subgroups included (1) protocol: vitamin C monotherapy compared to the vitamin C combination treatment group (vitamin C combined with thiamine and/or hydrocortisone); (2) the quality of the studies: low-quality studies compared with high-quality studies; (3) blind method: according to the blind method, the studies were divided into four groups: double-blind, single-blind, open-label, and unclear; and (4) publication year: according to the year of publication, the studies were divided into four groups: publication in and before 2019, in 2020, in 2021, and in 2022.

## 3. Results

### 3.1. Study Identification and Selection

**Figure 1:**
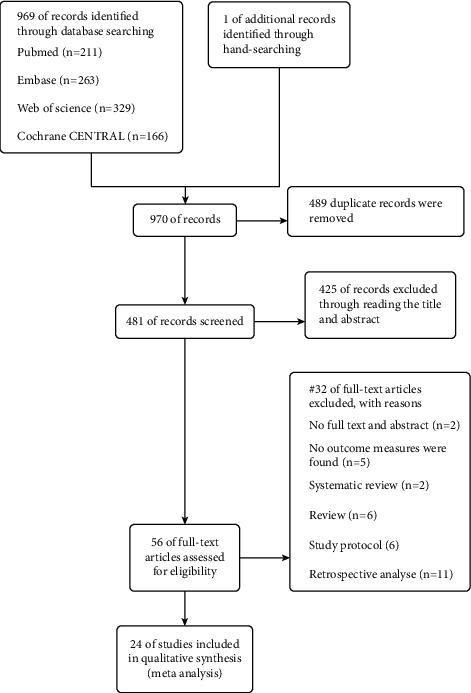
The selection flow diagram.

Initially, 969 pieces of literature were obtained from the aforementioned electronic databases, of which 211 were from PubMed, 263 from Embase, 329 from Web of Science, and 166 from Cochrane Central. In order to collect all relevant literature, one study was manually added after reading the relevant systematic review and meta-analysis. Eventually, a total of 970 studies were included in the screening. Of them, 489 duplicate records were removed, and 425 studies were omitted after reading the title and abstract, as they did not follow the inclusion criteria. Subsequently, we removed other 32 studies, including two with no full-text or abstract, five with no outcome measures, two systematic reviews, six reviews, six study protocols, and eleven retrospective studies. Finally, 24 eligible pieces of literature were included in the meta-analysis, with a total of 3,759 patients enrolled in the study [8, 10–17, 23–37]. The selection flow diagram is presented in Figure 1.

### 3.2. Study Characteristics

The characteristics of the 24 studies, including 23 full-text [8, 10–17, 23–35, 37] and 1 abstract [36], are shown in Table 1. Of those, ten studies were conducted in Asia [11, 12, 17, 23, 25, 30, 31, 35–37], nine in North America [8, 10, 13, 14, 16, 28, 29, 32, 33], three in Africa [24, 27, 34], and only one in Oceania. The study populations were mainly enriched in Asia and North America. In addition, 8 studies used vitamin C alone for the intervention in the treatment group [8, 10–13, 15–17], and the remaining 16 studies used vitamin C combined with thiamine and/or corticosteroids to treat patients [14, 23–37]. Nineteen studies were published in 2020 or later [12–17, 23, 24, 27–37], while only five were published before 2020 [8, 10, 11, 25, 26].

### 3.3. Results of Study Quality Assessment

**Figure 2:**
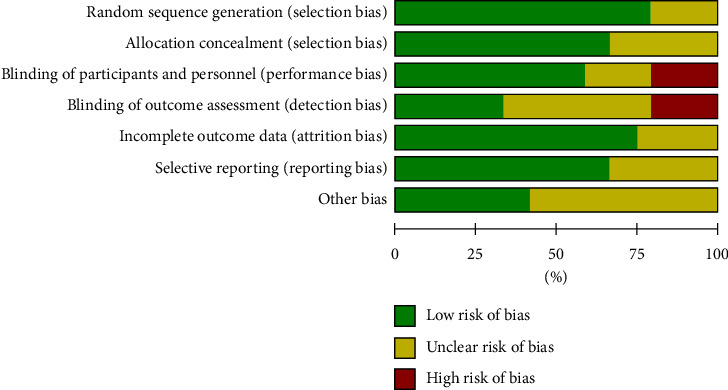
Risk assessment graph according to the Cochrane risk assessment tool.

Assessment of the quality of the study based on the Cochrane Collaboration's tool is shown in Figure 2, and the scores of the modified Jada scale are presented in Table 1. According to the Jada scores, most studies were of high quality [8, 10, 11, 13–17, 24, 26, 28–30, 32, 33, 35] and only eight were of low quality [12, 23, 25, 27, 31, 34, 36, 37].

### 3.4. Primary Outcome

#### 3.4.1. Overall Mortality

**Figure 3:**
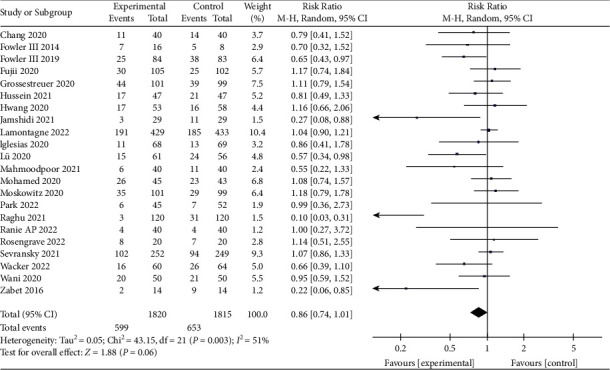
Forest plots for the effect of vitamin C on overall mortality.

**Figure 4:**
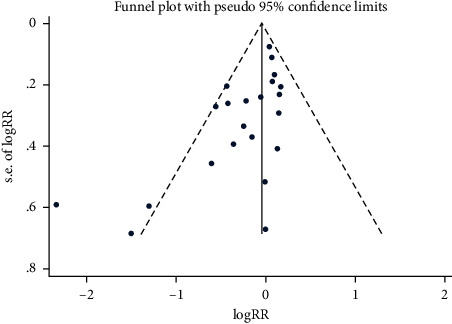
The funnel plot for publication bias, comparing the overall mortality in the vitamin C and control groups. A blue dot represents a single study, and the funnel dotted line represents 95% confidence intervals.

Twenty-two studies were included in our analysis [8, 10–17, 23, 24, 27–37], consisting of 3,635 patients, which included 1820 in the vitamin C treatment group and 1815 in the control group. The overall mortality in the vitamin C treatment group was lower than that in the control group, but the difference was not statistically significant (RR, 0.86; 95% CI, 0.74–1.01; *P*=0.06; *I*^2^ = 51%, Figure 3). We also found a high heterogeneity between the included studies; therefore, we conducted publication bias, meta-regression, subgroup, and sensitivity analyses to find the source of heterogeneity. The funnel plot (Figure 4) and Egger's test result (*P*=0.003) showed the apparent publication bias of the reports. Sensitivity analysis showed that the heterogeneity of the included work decreased significantly after removing the study by Raghu and Ramalingam [36]; however, the results did not change (RR, 0.92; 95% CI, 0.82–1.04; *P*=0.20; *I*^2^ = 26%, Supplementary Figure 1), which indicated the stability of the results. We also conducted a meta-regression analysis according to the protocol, quality of the studies, blinding method, and the publication year (Table 2). Subsequently, we performed a subgroup analysis based on the meta-regression results (Supplementary Figure 2).

### 3.5. Subgroup Analysis

#### 3.5.1. Protocol

We divided the studies into vitamin C monotherapy and combination treatment groups (Supplementary Figure 2A). The vitamin C monotherapy group included 8 studies [8, 10–13, 15–17], which showed that reduction in the overall mortality of the patients, but heterogeneity between the studies was significant (RR, 0.72; 95% CI, 0.54–0.96; *P*=0.03; *I*^2^ = 58%). On the other hand, the vitamin C combination treatment group [14, 23–37] did not show the same trend (RR, 0.94; 95% CI, 0.77–1.14; *P*=0.54; *I*^2^ = 49%). Furthermore, although the heterogeneity was evident between the two subgroups (*P*=0.13; *I*^2^ = 56.1%), it was not statistically significant.

#### 3.5.2. The Quality of the Studies

This analysis divided the studies into two subgroups: 7 low-quality studies [12, 23, 27, 31, 34, 36, 37] and 15 high-quality studies [8, 10, 11, 13–17, 24, 28–30, 32, 33, 35] (Supplementary Figure 2B). The low-quality studies revealed that vitamin C could reduce overall mortality (RR, 0.58; 95% CI, 0.36–0.93; *P*=0.03; *I*^2^ = 68%), while the high-quality studies did not show any reduction (RR, 0.98; 95% CI, 0.87–1.11; *P*=0.11; *I*^2^ = 19%). Moreover, the two subgroups showed statistically significant heterogeneity (*P*=0.04; *I*^2^ = 77.3%).

#### 3.5.3. Blind Method

We divided the studies into four subgroups: double-blind [8, 10, 11, 13–17, 28–30, 33, 35], single-blind [31], open-label [24, 27, 32, 36], and unclear [12, 23, 34, 37] groups (Supplementary Figure 2C). The double-blind (RR, 0.95; 95% CI, 0.82–1.10; *P*=0.48; *I*^2^ = 28%), single-blind (RR, 0.79; 95% CI, 0.41–1.52; *P*=0.47), and open-label (RR, 0.72; 95% CI, 0.37–1.40; *P*=0.34; *I*^2^ = 84%) groups indicated that vitamin C could not reduce the overall mortality of the patients. On the other hand, the unclear group showed a reduction in the overall mortality of the patients (RR, 0.65; 95% CI, 0.44–0.95; *P*=0.03; *I*^2^ = 16%). There was no significant heterogeneity among the subgroups (*P*=0.26; *I*^2^ = 24.4%).

#### 3.5.4. Publication Year

As most of the literature was published after 2019, we divided them into four subgroups: publication in and before 2019 [8, 10, 11], in 2020 [12, 14, 24, 27–32], in 2021 [23, 33, 36, 37], and in 2022 [13, 15, 16, 34, 35] (Supplementary Figure 2D). The studies published in and before 2019 (RR, 0.60; 95% CI, 0.39–0.92; *P*=0.02; *I*^2^ = 19%) and in 2021 (RR, 0.49; 95% CI, 0.24–0.99; *P*=0.05; *I*^2^ = 84%) indicated that vitamin C could reduce overall mortality of patients with sepsis, while the studies published in 2020 (RR, 1.02; 95% CI, 0.87–1.18; *P*=0.84; *I*^2^ = 0%) and 2022 (RR, 1.01; 95% CI, 0.88–1.16; *P*=0.06; *I*^2^ = 51%) showed a different result. Moreover, the subgroups showed statistically significant heterogeneity (*P*=0.03; *I*^2^ = 66.7%).

### 3.6. Secondary Outcomes

#### 3.6.1. 28-Day Mortality

**Figure 5:**
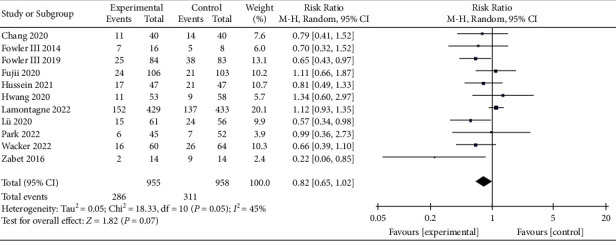
Forest plots for the effect of vitamin C on the 28-day mortality.

A total of 11 studies on 28-day mortality were included in the meta-analysis [8, 10–13, 16, 23, 30–32, 35], with 1,913 patients enrolled. Our analysis showed that patients who received vitamin C had a lower 28-day mortality than the control group (RR, 0.82; 95% CI, 0.65–1.02; *P*=0.07; *I*^2^ = 45%, Figure 5); however, the difference was not statistically significant. The funnel plot (Supplementary Figure 3) and Egger's test result (*P*=0.234) showed no publication bias of the included studies.

#### 3.6.2. 30-Day Mortality

**Figure 6:**
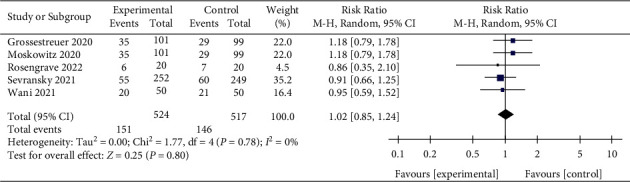
Forest plots for the effect of vitamin C on the 30-day mortality.

An analysis of five studies reporting 30-day mortality [14, 15, 27, 28, 33] showed that vitamin C did not improve the 30-day mortality of the patients (RR, 1.02; 95% CI, 0.85–1.24; *P*=0.80; *I*^2^ = 0%, Figure 6). Furthermore, there was no heterogeneity among the included studies, and the result was credible.

#### 3.6.3. 90-Day Mortality

**Figure 7:**
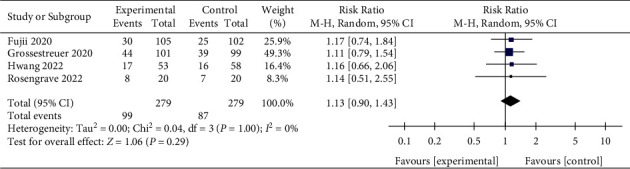
Forest plots for the effect of vitamin C on the 90-day mortality.

Only four studies published after 2020, with 558 patients, had reported 90-day mortality [14, 15, 30, 32]. Vitamin C did not reduce the 90-day mortality (RR, 1.13; 95% CI, 0.90–1.43; *P*=0.29; *I*^2^ = 0%, Figure 7). This result was credible, as there was no heterogeneity among the included studies.

#### 3.6.4. 180-Day Mortality

**Figure 8:**
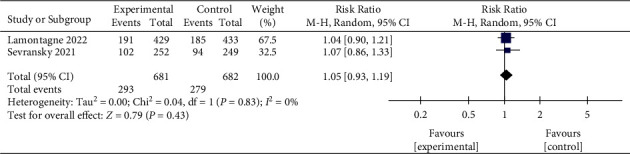
Forest plots for the effect of vitamin C on the 180-day mortality.

Two studies were included in this outcome [13, 33] with 572 patients enrolled. Vitamin C did not reduce the 180-day mortality of patients with sepsis (RR, 1.05; 95% CI, 0.93–1.19; *P*=0.43; *I*^2^ = 0%, Figure 8).

### 3.7. ICU Mortality

**Figure 9:**
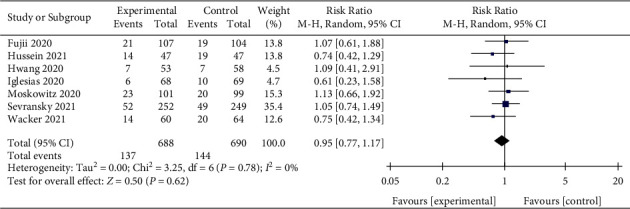
Forest plots for the effect of vitamin C on ICU mortality.

Seven studies, published after 2020, reported ICU mortality [16, 23, 28–30, 32, 33]. Although the results showed a slight reduction in the ICU mortality of patients with sepsis with IV vitamin C, the difference was not statistically significant (RR, 0.95; 95% CI, 0.77–1.17; *P*=0.62; *I*^2^ = 0%, Figure 9).

### 3.8. Hospital Mortality

**Figure 10:**
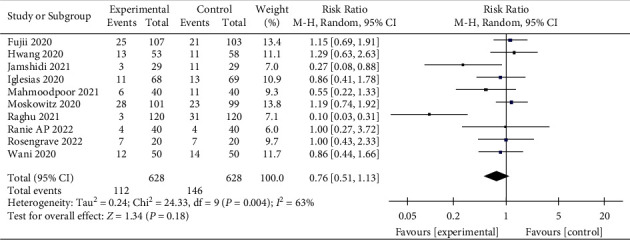
Forest plots for the effect of vitamin C on hospital mortality.

Ten studies reported hospital mortality with 1,256 patients enrolled [15, 17, 27–30, 32, 34, 36, 37]. Although vitamin C marginally reduced hospital mortality (RR, 0.76; 95% CI, 0.51–1.13; *P*=0.18; *I*^2^ = 63%), the difference was not statistically significant (Figure 10). Moreover, there was significant heterogeneity in the included studies. We also generated funnel plots (Supplementary Figure 4) and performed the Egger test (*P*=0.024) to detect publication bias. Our analysis demonstrated a significant publication bias in the included studies. Therefore, we further conducted a sensitivity analysis. When we removed the study of Raghu et al., the heterogeneity of the included studies was significantly reduced, but the results remained the same (RR, 0.97; 95% CI, 0.761–1.23; *P*=0.79; *I*^2^ = 2%, Supplementary Figure 5), demonstrating the stability of the obtained results.

### 3.9. New Onset of AKI

**Figure 11:**
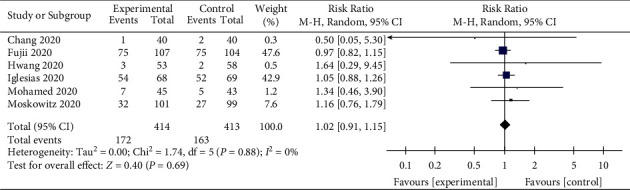
Forest plots for the effects of vitamin C on the new onset of AKI.

Acute renal injury caused by sepsis is widespread. Six studies reported the outcome of the new onset of AKI [24, 28–32]. However, the treatment of sepsis with vitamin C could not reduce the incidence of AKI in the patients (RR, 1.02; 95% CI, 0.91–1.15; *P*=0.69; *I*^2^ = 0%, Figure 11).

### 3.10. Adverse Events

**Figure 12:**
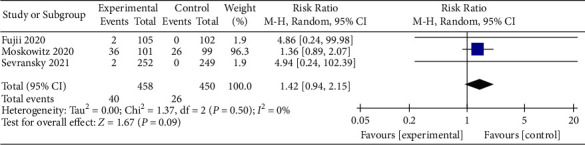
Forest plots for the effects of vitamin C on the adverse event.

As we should also pay attention to the safety and effectiveness of the treatment, we searched the literature for evidence of adverse events. The adverse events included hyperglycemia, hypernatremia, hospital-acquired infection, fluid over-load and hyperglycemia, hemorrhagic shock, and worsening kidney function. Only 3 literature were included in the study [28, 32, 33]. The incidence of adverse events in the vitamin C treatment group was higher than that in the control group (RR, 1.42; 95% CI, 0.94–2.15; *P*=0.09; *I*^2^ = 0%, Figure 12); however, the difference was not statistically significant.

### 3.11. Duration of Vasopressors

**Figure 13:**
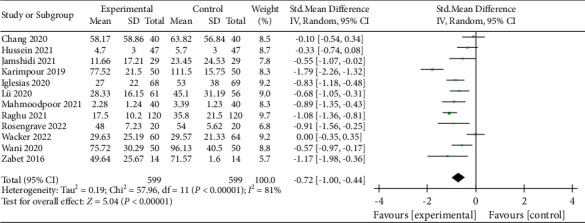
Forest plots for the effect of vitamin C on the duration of vasopressors.

Twelve studies reported the outcome of the duration of vasopressors, and a total of 1198 patients were included [11, 12, 15–17, 23, 26, 27, 29, 31, 36, 37]. The analysis of the included studies showed that vitamin C could significantly reduce the duration of vasopressors, but the two groups demonstrated significant heterogeneity (RR, −0.72; 95% CI, −1.00–−0.44; *P* < 0.00001; *I*^2^ = 81%, Figure 13). There was no significant publication bias in the included studies (Supplementary Figure 6), and the Egger test (*P*=0.892) also showed the same result. Sensitivity analysis showed that the heterogeneity of the included literature was not significantly changed when any one study was removed, and the result remained changed (Supplementary Figure 7).

### 3.12. Duration of Mechanical Ventilation

**Figure 14:**
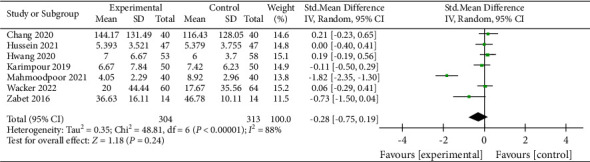
Forest plots for the effect of vitamin C on the duration of mechanical ventilation.

A total of seven studies reported this outcome [11, 16, 17, 23, 25, 30, 31]. The result showed that vitamin C could reduce the duration of mechanical ventilation (RR, −0.28; 95% CI, −0.75–0.19; *P*=0.24; *I*^2^ = 88%, Figure 14). Significant heterogeneity existed in the included studies, and due to the small number of included studies, we could not detect publication bias. After sensitivity analysis, we found that only when the report from Mahmoodpoor et al. [17] was removed, the heterogeneity of the included studies significantly decreased, but the result also altered (RR, 0.03; 95% CI, −0.14–0.20; _*P*=0.74_; *I*^2^ = 11, Supplementary Figure 8). Therefore, we considered that the result was unstable.

### 3.13. ∆SOFA (72 h)

**Figure 15:**
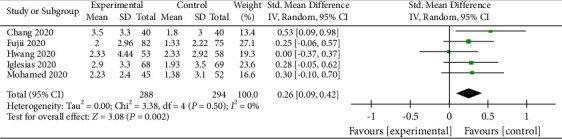
Forest plots for the effect of vitamin C on ∆SOFA (72 h).

∆SOFA (72 h) represents the change in SOFA score over 72 hours of intervention. Only five studies reported this outcome [24, 29–32]. Vitamin C significantly decreased SOFA score compared with the control group (RR, 0.26; 95% CI, 0.09–0.42; *P*=0.002; *I*^2^ = 0%, Figure 15). Moreover, the results were credible.

### 3.14. Length of ICU Stay

**Figure 16:**
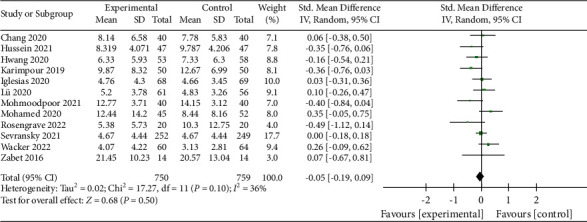
Forest plots for the effect of vitamin C on length of ICU stay.

Twelve reports and 1,509 patients were included in the analysis regarding the length of ICU stay [11, 12, 15–17, 23–25, 29–31, 33]. Results suggested that vitamin C could reduce the length of ICU stay (RR, −0.05; 95% CI, −0.19–0.09; *P*=0.50; *I*^2^ = 36%, Figure 16). However, the reduction was not statistically significant, and the result was credible. The funnel plot (Supplementary Figure 9) and the Egger test (*P*=0.339) showed no significant publication bias in the included studies.

### 3.15. Length of Hospital Stay

**Figure 17:**
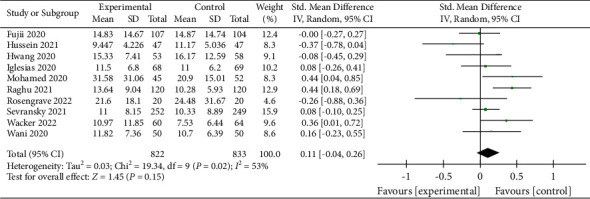
Forest plots for the effect of vitamin C on length of hospital stay.

Ten pieces of literature reported the outcome of the length of hospital stay [15, 16, 23, 24, 27, 29, 30, 32, 33, 36]. Our analysis showed that vitamin C did not significantly reduce the length of the hospital stay (RR, 0.11; 95% CI, −0.04–0.26; *P*=0.15; *I*^2^ = 53%, Figure 17). Moreover, the included studies exhibited significant heterogeneity. The funnel plot (Supplementary Figure 10) and Egger test (*P*=0.630) showed no significant publication bias in the included studies. Moreover, sensitivity analysis showed that when only the study by Raghu et al. was removed, the heterogeneity changed significantly. However, the result remained unchanged (Supplementary Figure 11), indicating the stability of the results.

## 4. Discussion

We performed a systematic review and meta-analysis to evaluate the efficacy and safety of IV vitamin C in the treatment of sepsis. This meta-analysis has the largest number of included literature and cases to date. The primary outcome analysis showed that IV vitamin C tended to reduce overall mortality in patients with sepsis compared with the control group. However, the difference between the two groups was not statistically significant (Figure 3), which is consistent with the result of Patel et al. [38].

The heterogeneity among the included studies was significant. Results of statistically significant studies are more likely to be reported and published than nonsignificant and invalid results, which may lead to publication bias. The included studies had significant publication bias, as shown by the funnel plot (Figure 4) and the Egger tests (*P*=0.003). In the sensitivity analysis, the heterogeneity was significantly decreased after excluding the report by Raghu and Ramalingam [36] with a significant publication bias; the *I*^2^ decreased from 51% to 26%, but the outcome remained unchanged. After removing the three reports [11, 36, 37] with possible publication bias, the result remained unchanged, with *I*^2^ decreasing from 51% to 0% (Supplementary Figure 12). These results indicate that vitamin C could improve overall mortality in patients with sepsis. We also performed the meta-regression based on the quality of studies, study protocol, blinding method, and publication year, and the *P* values were 0.013, 0.027, 0.746, and 0.053, respectively. The meta-regression and subgroup analysis showed that the quality of studies (*P*=0.04; *I*^2^ = 77.3%) could be the primary source of the heterogeneity, while the protocol (*P*=0.13; *I*^2^ = 56.1%) and the publication year (*P*=0.03; *I*^2^ = 66.7%) could also contribute to the heterogeneity. Consistent with Patel et al. [38], our subgroup analysis suggested that compared with the combination treatment group, vitamin C monotherapy showed a decreasing trend in overall mortality. However, it differed from the result of a high-quality randomized controlled study published in 2022 by Lamontagne et al. [13]. Their results indicated that vitamin C monotherapy did not reduce overall mortality. Therefore, the efficacy of vitamin C monotherapy in reducing overall mortality is uncertain and needs to include a large number of high-quality studies. Furthermore, low-quality studies indicated that vitamin C could improve the overall mortality of patients with sepsis, while high-quality studies did not show the same trend. Low-quality studies were mainly published in 2021. In the subgroup of years of publication, only the studies published in 2021 showed that vitamin C reduced overall mortality in patients with sepsis; the literature published in other time groups did not show the same trend. In the blind method subgroups, the unclear group showed an exciting result, but the scores of the included studies were less than or equal to 3. In other words, all the included studies were of low quality, indicating that low-quality literature can significantly affect the bias in outcomes. The finding that vitamin C could improve overall mortality in patients with sepsis was stable, but the results of recently published high-quality studies did not show the same trend [13, 33]. According to the GRADE system [39], the evidence that vitamin C could improve overall mortality in patients with sepsis was of moderate quality.

The 28-day and 30-day mortalities were defined as short-term mortality, while 90-day and 180-day mortalities were defined as long-term mortality. IV vitamin C tended to reduce 28-day mortality in patients with sepsis, while the 30-day mortality remained unaffected. Vitamin C also showed no efficacy in decreasing 90-day and 180-day mortalities. Therefore, our result suggested that vitamin C did not improve the long-term mortality of sepsis. In addition, we also found that vitamin C improves ICU and hospital mortalities, but the difference was not statistically significant compared with the control group. As sepsis is an acute disease, most patients' ICU and hospital stays are relatively short (generally less than 30 days), consistent with our included studies. Therefore, we can conclude that vitamin C might play a role in improving short-term mortality. This result is consistent with the meta-analysis of Li et al. [40].

The SOFA score is used to evaluate organ function damage in patients with sepsis [41]. It is the sum of respiratory status, liver function, renal function, coagulation function, circulatory status, and nervous system score. The SOFA score decreases with the improvement in organ functions; therefore, the △SOFA score would have a positive value. We demonstrated that vitamin C treatment for over 72 hours in patients with sepsis could improve their organ damage. This is consistent with the results of the meta-analysis published in 2021 and 2022 [40, 42]. However, vitamin C did not show a positive trend for reducing the occurrence of AKI. Vitamin C might reduce SOFA score by improving the coagulation function and circulatory status and reducing inflammatory response [7].

Our results showed that vitamin C could reduce the length of ICU stay, while the hospital stay did not indicate the same trend. All these differences, however, were not statistically significant. The included literature was highly heterogeneous regarding the duration of vasopressors and mechanical ventilation; therefore, the results were not credible.

The incidence of adverse reactions in the vitamin C treatment group was comparable to the control group. Major adverse effects included hyperglycemia, hypernatremia, fluid overload, and hospital-acquired infection. The reports concerning these adverse events used a combination of vitamin C with thiamine and hydrocortisone [28, 32, 33]. The largest number of adverse events was reported by Moskowitz et al. [28], with vitamin C combined with thiamine and hydrocortisone in the intervention group and a placebo in similar volumes (0.9% sodium chloride) in the control group. Hyperglycemia, hypernatremia, and fluid overload are adverse reactions of hydrocortisone [43, 44], while no adverse effects have been reported with vitamin C monotherapy. Moreover, the occurrence of hospital-acquired infection in the treatment and control groups was comparable [28]. There is no substantial evidence that IV vitamin C increases the incidence of adverse events in patients with sepsis.

### 4.1. The Strength and Limitations

There are many strengths of this research. Our study was conducted according to our previously developed protocol published in PROSPERO. We conducted a comprehensive literature search to avoid bias as much as possible. We also examined the sources of heterogeneity in the main study results. Moreover, the design types of the included literature stipulated that only RCTs were included, and further inclusion and exclusion criteria were formulated. However, there are still many limitations to our study. First, the inclusion of low-quality literature might have affected the results; the heterogeneity was evident, which may have affected the credibility of the results. Second, the included studies varied in intervention and the dosages of vitamin C and the regional distribution and ethnic group of the enrolled populations varied, which may increase sources of heterogeneity. Third, due to different follow-up time, the outcome measures are not exactly the same, which may also be the source of heterogeneity. Fourth, due to different follow-up time, we could not analyze the effect of IV vitamin C on the time of free vasopressors, mechanical ventilation, and ICU stay. Finally, although some of our outcomes, such as overall mortality, 28-day mortality, and length of ICU stay, showed positive effects, 95% confidence intervals intersected the null line, which may impact interpretation of the results.

## 5. Conclusion

Our analysis showed that intravenous vitamin C might improve short-term mortality and overall mortality of sepsis. Moreover, the SOFA score of patients with sepsis improved significantly after treatment with vitamin C over 72 hours. The main results of our study were moderate-quality evidence. More high-quality, multicenter RCTs are needed to provide stronger evidence on the efficacy and safety of IV vitamin C for patients with sepsis.

## Figures and Tables

**Table 1:** Characteristics of the included studies.

First author	Year	Nation	Jada score	Study design	Patients (treatment/control)	Treatment intervention	Control intervention
Hussein	2021	Egypt	2	SC, unclear, RCT	94 (47/47)	Hydrocortisone 50 mg/6 hours IV for 7 days or till ICU discharge, followed by tapering; vitamin C 1.5 g/6 hours IV for 4 days or till ICU discharge; and thiamine 200 mg/12 hours IV for 4 days or till ICU discharge	Hydrocortisone 50 mg/6 hours IV for 7 days or till intensive care unit (ICU) discharge
Mohamed	2020	India	4	SC, open-label, RCT	88 (45/43)	Vitamin C (1.5 g every 6 hours), thiamine (200 mg every 12 hours), and hydrocortisone (50 mg every 6 hours) for at least 4 days	Thiamine (200 mg every 12 hours) and hydrocortisone (50 mg every 6 hours) for at least 4 days
Fowler III	2014	USA	6	SC, DB, RCT	24 (16/8)	(Lo-AscA):50 mg/kg/24 hours (8 patients)	Placebo (5% dextrose in water)
						Hi-(AscA): 200 mg/kg/24 hours (8 patients)	
Rosengrave	2022	New Zealand	6	SC, DB, RCT	40 (20/20)	Vitamin C (25 mg/kg/h)	Placebo (5% dextrose in water)
Wacker	2022	USA	4	MC, DB, RCT	124 (60/64)	1000 mg bolus of vitamin C over 30 minutes, followed by a continuous infusion of 250 mg/h for 96 hours	Placebo (normal saline)
Mahmoodpoor	2021	Iran	6	SC, DB, RCT	80 (40/40)	60 mg/kg/day vitamin C for 96 hours	Normal saline for 96 hours
Karimpour	2019	Iran	3	SC, SB, RCT	100 (50/50)	Vitamin C at a dose of 50 mg/kg/four times daily along with thiamine at a dose of 200 mg/twice daily	Placebo (normal saline for four days)
Balakrishnan	2018	India	6	SC, SB, RCT	24 (12/12)	6 g vitamin C, 400 mg thiamine, and 200 mg hydrocortisone per day for 4 days	Placebo
Wani	2020	India	1	SC, open-label, RCT	100 (50/50)	Vitamin C (1.5 grams every 6 hourly for 4 days or until discharge from the hospital), hydrocortisone (50 mg every 6 hourly for 7 days or until ICU discharge followed by a taper over 3 days), and intravenous thiamine (200 mg *q* 12 hourly for 4 days or until discharge from the hospital) +standard therapy	Standard therapy alone
Moskowitz	2020	USA	7	MC, DB, RCT	200 (101/99)	Vitamin C (1500 mg), hydrocortisone (50 mg), and thiamine (100 mg) every 6 hours for 4 days	Placebo (0.9% sodium chloride)
Lglesias	2020	USA	7	MC, DB, RCT	137 (68/69)	1,500 mg of vitamin C every 6 hours, 200 mg of thiamine every 12 hours, and 50 mg of hydrocortisone every 6 hours for 4 days	Placebo (saline)
Hwang	2020	Korea	7	MC, DB, RCT	111 (53/58)	Vitamin C (50 mg/kg, maximum single dose 3 g, daily dose 6 g) and thiamine (200 mg) were mixed in a 50-ml 0.9% saline bag, respectively, and intravenously administered to patients over 60 min every 12 hours for a total of 48 hours	Placebo (0.9% saline)
Chang	2020	China	3	SC, SB, RCT	80 (40/40)	Hydrocortisone (50 mg every 6 hours for 7 days), vitamin C (1.5 g every 6 hours for 4 days), and thiamine (200 mg every 12 hours for 4 days)	Placebo (normal saline)
Fujii	2020	USA	5	MC, open-label, RCT	216 (107/104)	Vitamin C (1.5 g every 6 hours), hydrocortisone (50 mg every 6 hours), and thiamine (200 mg every 12 hours)	Hydrocortisone (50 mg every 6 hours) alone until shock resolution or up to 10 days
Sevransky	2021	USA	7	MC, DB, RCT	501 (252/249)	Vitamin C (1.5 g), thiamine (100 mg), and hydrocortisone (50 mg) every 6 hours for 4 days or until ICU discharge	Placebo
Fowler III	2019	USA	7	MC, DB, RCT	167 (84/83)	Vitamin C (50 mg/kg in dextrose 5% in water for 96 hours	Placebo (5% dextrose in water)
Zabet	2016	Iran	4	SC, DB, RCT	28 (14/14)	25 mg/kg intravenous vitamin C every 6 hours for 72 hours	Placebo (5% dextrose in water)
Lü	2020	China	3	SC, unclear, RCT	117 (61/56)	3.0 g vitamin C dissolved into 5% dextrose (100 ml/time, 2 times/day) until ICU discharge	Placebo 5% dextrose (100 ml/time, 2 times/day)
Lamontagne	2022	Canada	7	MC, DB, RCT	867 (429/433)	Vitamin C (at a dose of 50 mg per kg of body weight), every 6 hours for 96 hours	Placebo
Ranie AP	2022	India	3	SC, unclear, RCT	80 (40/40)	Vitamin C (2 g every 8 hours) or vitamin C + thiamine (200 mg 12 hourly)	Thiamine (200 mg 12 hourly) or none
Park	2022	Korea	6	MC, DB, RCT	97 (45/52)	Vitamin C (50 mg/kg, maximum single dose: 3 g mixed with 50 mL of normal saline) and thiamine (200 mg mixed with 50 mL of normal saline) were administered every 12 hours to the treatment group for a total of 48 hours	Placebo (saline)
Grossestreuer	2020	USA	4	MC, DB, RCT	200 (101/99)	Vitamin C (1,500 mg), hydrocortisone (50 mg), and thiamine (100 mg) every 6 hours for 4 days	Placebo
Raghu	2021	Malaysia	2	Unclear, open-label, RCT	240 (120/120)	Standard care with hydrocortisone, vitamin C, and thiamine	Standard care
Jamshidi	2021	Iran	3	SC, unclear, RCT	58 (29/29)	Hydrocortisone (50 mg/6 h), vitamin C (1.5 g/6 h), and thiamine (200 mg/12 h)	Routine care

**Table 2:** Meta-regression analysis summary according to the protocol, quality of the studies, blinding method, and publication year.

	Coeff.	Std. err.	*z*	*P* > |*z*|	95% conf. interval
Protocol	0.2654378	0.1070116	2.48	0.013	0.0556989 0.4751767

Studies' quality	0.4563622	0.2063868	2.21	0.027	0.0518515 0.8608729

Blind method	0.0242355	0.0747274	0.32	0.746	−0.1222275 0.1706986

Publication year	0.1049809	0.0542003	1.94	0.053	−0.0012498 0.2112116

## Data Availability

The data used to support the findings of this study are available from the corresponding author upon request.

## Supplementary Materials

Supplementary Materials Supplementary Figure 1: Forest plots for the vitamin C effect on the overall mortality after removing the study of Raghu et al. Supplementary Figure 2: Forest plots for subgroup analysis of overall mortality according to protocol (A), the quality of the studies (B), blind method (C), and publication year (D). Supplementary Figure 3: Funnel plot for publication bias, which compared the 28-day mortality in the vitamin C group and control group. Supplementary Figure 4: Funnel plot for publication bias, which compared the hospital mortality in the vitamin C group and control group. Supplementary Figure 5: Forest plots for the vitamin C effect on the hospital mortality after removing the study of Raghu et al. Supplementary Figure 6: Funnel plot for publication bias, which compared the vasopressors duration in the vitamin C group and control group. Supplementary Figure 7: Sensitivity analysis for vasopressors duration. Supplementary Figure 8: Sensitivity analysis for duration of mechanical ventilation. After removal of the study of Mahmoodpoor et al. [17], the heterogeneity of the included studies significantly decreased, and the result was also changed (E). Supplementary Figure 9: Funnel plot for publication bias, which compared the length of ICU stay in the vitamin C group and control group. Supplementary Figure 10: Funnel plot for publication bias which compared the hospital stay in the vitamin C group and control group. Supplementary Figure 11: Sensitivity analysis for length of hospital stay. After removal of the study of Raghu et al., the heterogeneity changed significantly, but the result did not change (F). Supplementary Figure 12: Forest plots for the vitamin C effect on the overall mortality after removing the study of Jamshidi et al., Raghu et al., and Zabet et al.
