# Supplementary material for: IV Vitamin C in Sepsis: A Latest Systematic Review and Meta-Analysis
Source: Int J Clin Pract. 2023 Jan 24;2023:6733465. doi: 10.1155/2023/6733465 (PMC9889164; doi:10.1155/2023/6733465)
Supplement: Supplementary Materials — Supplementary Figure 1: Forest plots for the vitamin C effect on the overall mortality after removing the study of Raghu et al. Supplementary Figure 2: Forest plots for subgroup analysis of overall mortality according to protocol (A), the quality of the studies (B), blind method (C), and publication year (D). Supplementary Figure 3: Funnel plot for publication bias, which compared the 28-day mortality in the vitamin C group and control group. Supplementary Figure 4: Funnel plot for publication bias, which compared the hospital mortality in the vitamin C group and control group. Supplementary Figure 5: Forest plots for the vitamin C effect on the hospital mortality after removing the study of Raghu et al. Supplementary Figure 6: Funnel plot for publication bias, which compared the vasopressors duration in the vitamin C group and control group. Supplementary Figure 7: Sensitivity analysis for vasopressors duration. Supplementary Figure 8: Sensitivity analysis for duration of mechanical ventilation. After removal of the study of Mahmoodpoor et al. [17], the heterogeneity of the included studies significantly decreased, and the result was also changed (E). Supplementary Figure 9: Funnel plot for publication bias, which compared the length of ICU stay in the vitamin C group and control group. Supplementary Figure 10: Funnel plot for publication bias which compared the hospital stay in the vitamin C group and control group. Supplementary Figure 11: Sensitivity analysis for length of hospital stay. After removal of the study of Raghu et al., the heterogeneity changed significantly, but the result did not change (F). Supplementary Figure 12: Forest plots for the vitamin C effect on the overall mortality after removing the study of Jamshidi et al., Raghu et al., and Zabet et al. [file 6733465.f1.zip › supplementary figure 2.pdf]

Study or Subgroup	Experimental Events	Experimental Total	Control Events	Control Total	Weight	Risk Ratio M-H, Random, 95% CI
**1.2.1 vitamin C alone**						
Fowler III 2014	7	16	5	8	2.9%	0.70 [0.32, 1.52]
Fowler III 2019	25	84	38	83	6.4%	0.65 [0.43, 0.97]
Lamontagne 2022	191	429	185	433	10.4%	1.04 [0.90, 1.21]
Lv 2020	15	61	24	56	4.8%	0.57 [0.34, 0.98]
Mahmoodpoor 2021	6	40	11	40	2.4%	0.55 [0.22, 1.33]
Rosengrave 2022	8	20	7	20	2.8%	1.14 [0.51, 2.55]
Wacker 2022	16	60	26	64	5.0%	0.66 [0.39, 1.10]
Zabet 2016	2	14	9	14	1.2%	0.22 [0.06, 0.85]
**Subtotal (95% CI)**		**724**		**718**	**35.9%**	**0.72 [0.54, 0.96]**
Total events	270		305			
Heterogeneity: Tau² = 0.08; Chi² = 16.59, df = 7 (P = 0.02); I² = 58%						
Test for overall effect: Z = 2.23 (P = 0.03)						
**1.2.2 vitamin C combined with thiamine and/or corticosteroids**						
Chang 2020	11	40	14	40	3.7%	0.79 [0.41, 1.52]
Fujii 2020	30	105	25	102	5.7%	1.17 [0.74, 1.84]
Grossestreuer 2020	44	101	39	99	7.5%	1.11 [0.79, 1.54]
Hussein 2021	17	47	21	47	5.2%	0.81 [0.49, 1.33]
Hwang 2020	17	53	16	58	4.4%	1.16 [0.66, 2.06]
Jamshidi 2021	3	29	11	29	1.5%	0.27 [0.08, 0.88]
Iglesias 2020	11	68	13	69	3.2%	0.86 [0.41, 1.78]
Mohamed 2020	26	45	23	43	6.8%	1.08 [0.74, 1.57]
Moskowitz 2020	35	101	29	99	6.4%	1.18 [0.79, 1.78]
Park 2022	6	45	7	52	1.9%	0.99 [0.36, 2.73]
Raghu 2021	3	120	31	120	1.5%	0.10 [0.03, 0.31]
Ranie AP 2022	4	40	4	40	1.2%	1.00 [0.27, 3.72]
Sevransky 2021	102	252	94	249	9.3%	1.07 [0.86, 1.33]
Wani 2020	20	50	21	50	5.5%	0.95 [0.59, 1.52]
**Subtotal (95% CI)**		**1096**		**1097**	**64.1%**	**0.94 [0.77, 1.14]**
Total events	329		348			
Heterogeneity: Tau² = 0.06; Chi² = 25.31, df = 13 (P = 0.02); I² = 49%						
Test for overall effect: Z = 0.61 (P = 0.54)						
**Total (95% CI)**		**1820**		**1815**	**100.0%**	**0.86 [0.74, 1.01]**
Total events	599		653			
Heterogeneity: Tau² = 0.05; Chi² = 43.15, df = 21 (P = 0.003); I² = 51%						
Test for overall effect: Z = 1.88 (P = 0.06)						
Test for subgroup differences: Chi² = 2.28, df = 1 (P = 0.13), I² = 56.1%						

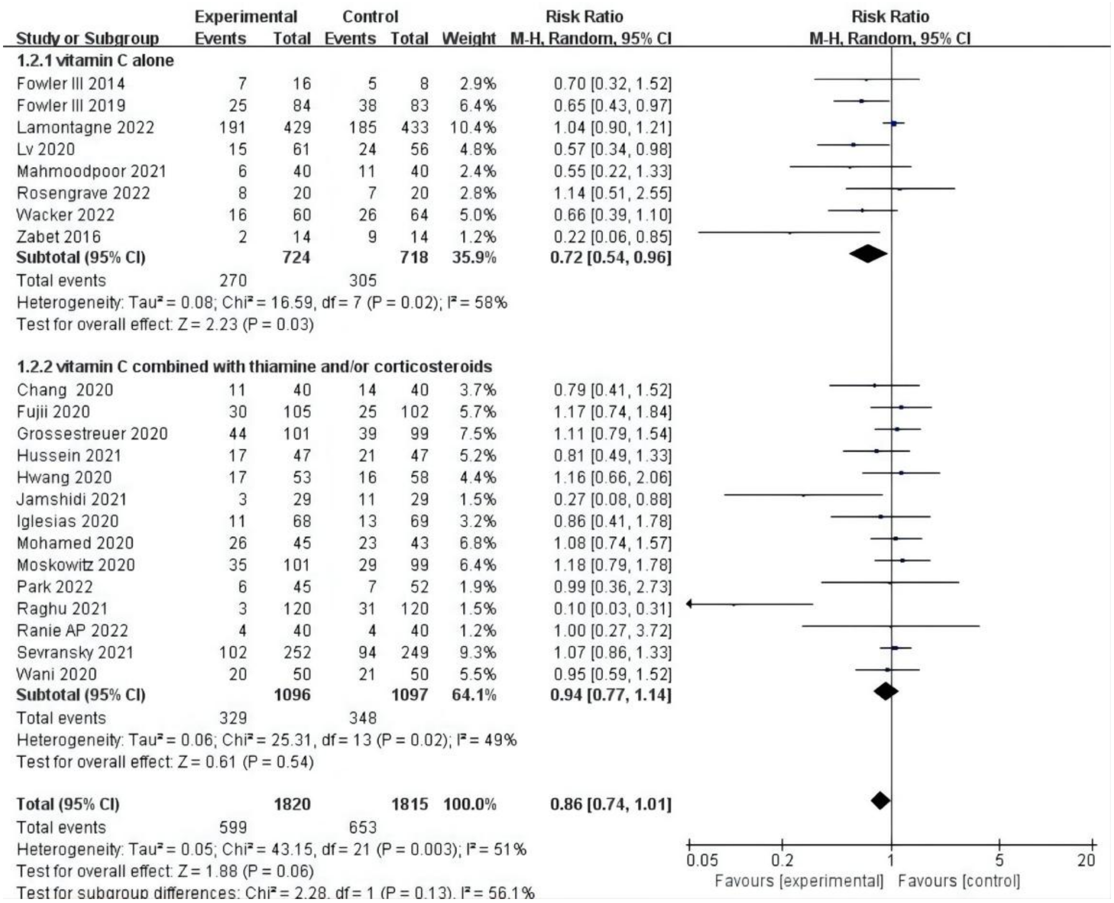

A

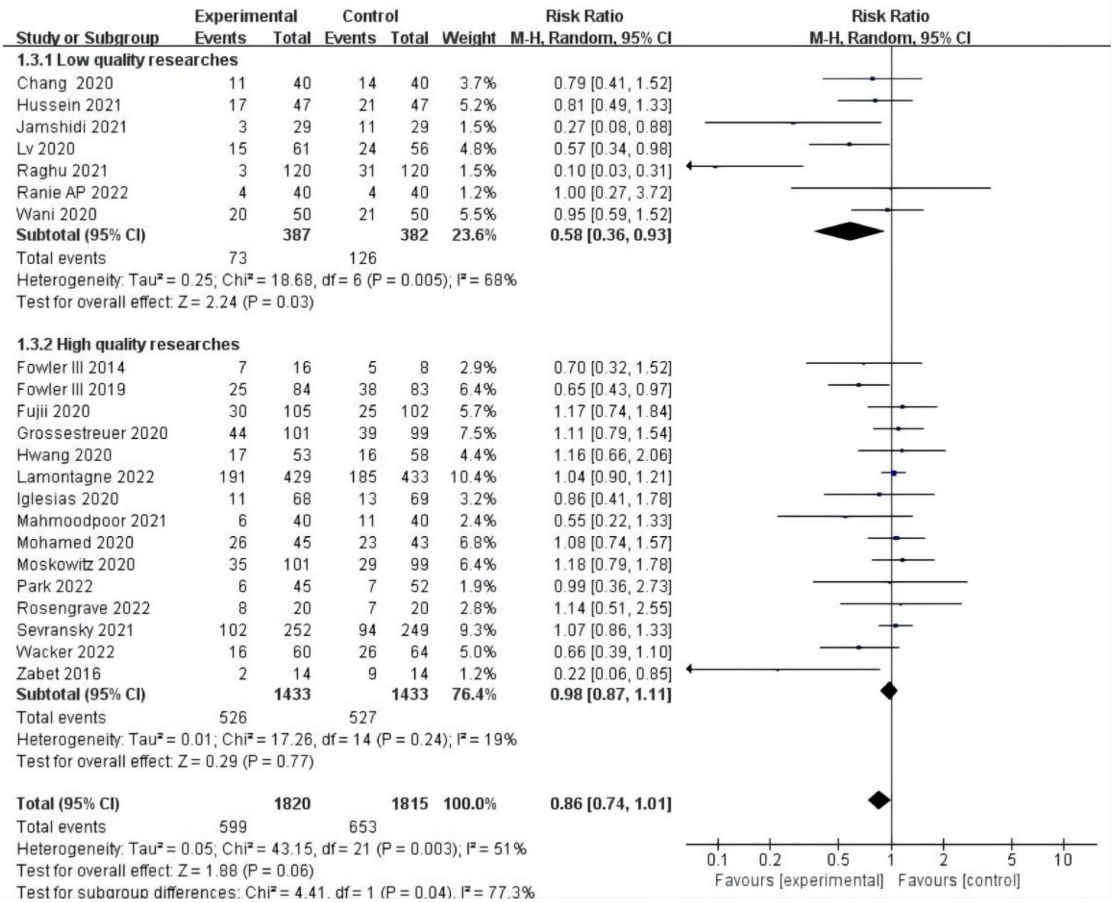
Experimental
Control
Risk Ratio
Risk Ratio
Study or Subgroup Events Total Events Total Weight M-H, Random, 95% CI
M-H, Random, 95% CI
1.3.1 Low quality researches
Chang 2020 11 40 14 40 3.7% 0.79 [0.41, 1.52]
Hussein 2021 17 47 21 47 5.2% 0.81 [0.49, 1.33]
Jamshidi 2021 3 29 11 29 1.5% 0.27 [0.08, 0.88]
Lv 2020 15 61 24 56 4.8% 0.57 [0.34, 0.98]
Raghu 2021 3 120 31 120 1.5% 0.10 [0.03, 0.31]
Ranie AP 2022 4 40 4 40 1.2% 1.00 [0.27, 3.72]
Wani 2020 20 50 21 50 5.5% 0.95 [0.59, 1.52]
Subtotal (95% CI) 387 382 23.6% 0.58 [0.36, 0.93]
Total events 73 126
Heterogeneity: Tau² = 0.25; Chi² = 18.68, df = 6 (P = 0.005); I² = 68%
Test for overall effect: Z = 2.24 (P = 0.03)
1.3.2 High quality researches
Fowler III 2014 7 16 5 8 2.9% 0.70 [0.32, 1.52]
Fowler III 2019 25 84 38 83 6.4% 0.65 [0.43, 0.97]
Fujii 2020 30 105 25 102 5.7% 1.17 [0.74, 1.84]
Grossestreuer 2020 44 101 39 99 7.5% 1.11 [0.79, 1.54]
Hwang 2020 17 53 16 58 4.4% 1.16 [0.66, 2.06]
Lamontagne 2022 191 429 185 433 10.4% 1.04 [0.90, 1.21]
Iglesias 2020 11 68 13 69 3.2% 0.86 [0.41, 1.78]
Mahmoodpoor 2021 6 40 11 40 2.4% 0.55 [0.22, 1.33]
Mohamed 2020 26 45 23 43 6.8% 1.08 [0.74, 1.57]
Moskowitz 2020 35 101 29 99 6.4% 1.18 [0.79, 1.78]
Park 2022 6 45 7 52 1.9% 0.99 [0.36, 2.73]
Rosengrave 2022 8 20 7 20 2.8% 1.14 [0.51, 2.55]
Sevransky 2021 102 252 94 249 9.3% 1.07 [0.86, 1.33]
Wacker 2022 16 60 26 64 5.0% 0.66 [0.39, 1.10]
Zabet 2016 2 14 9 14 1.2% 0.22 [0.06, 0.85]
Subtotal (95% CI) 1433 1433 76.4% 0.98 [0.87, 1.11]
Total events 526 527
Heterogeneity: Tau² = 0.01; Chi² = 17.26, df = 14 (P = 0.24); I² = 19%
Test for overall effect: Z = 0.29 (P = 0.77)
Total (95% CI) 1820 1815 100.0% 0.86 [0.74, 1.01]
Total events 599 653
Heterogeneity: Tau² = 0.05; Chi² = 43.15, df = 21 (P = 0.003); I² = 51%
Test for overall effect: Z = 1.88 (P = 0.06)
Test for subgroup differences: Chi² = 4.41, df = 1 (P = 0.04), I² = 77.3%
0.1 0.2 0.5 1 2 5 10
Favours [experimental] Favours [control]

B

Study or Subgroup	Experimental Events	Experimental Total	Control Events	Control Total	Weight	Risk Ratio M-H, Random, 95% CI
**1.12.1 Double-blind**						
Fowler III 2014	7	16	5	8	2.9%	0.70 [0.32, 1.52]
Fowler III 2019	25	84	38	83	6.4%	0.65 [0.43, 0.97]
Grossestreuer 2020	44	101	39	99	7.5%	1.11 [0.79, 1.54]
Hwang 2020	17	53	16	58	4.4%	1.16 [0.66, 2.06]
Lamontagne 2022	191	429	185	433	10.4%	1.04 [0.90, 1.21]
Iglesias 2020	11	68	13	69	3.2%	0.86 [0.41, 1.78]
Mahmoodpoor 2021	6	40	11	40	2.4%	0.55 [0.22, 1.33]
Moskowitz 2020	35	101	29	99	6.4%	1.18 [0.79, 1.78]
Park 2022	6	45	7	52	1.9%	0.99 [0.36, 2.73]
Rosengrave 2022	8	20	7	20	2.8%	1.14 [0.51, 2.55]
Sevransky 2021	102	252	94	249	9.3%	1.07 [0.86, 1.33]
Wacker 2022	16	60	26	64	5.0%	0.66 [0.39, 1.10]
Zabet 2016	2	14	9	14	1.2%	0.22 [0.06, 0.85]
**Subtotal (95% CI)**		**1283**		**1288**	**63.9%**	**0.95 [0.82, 1.10]**
Total events	470		479			
Heterogeneity: Tau² = 0.02; Chi² = 16.64, df = 12 (P = 0.16); I² = 28%						
Test for overall effect: Z = 0.70 (P = 0.48)						
**1.12.2 Single-blind**						
Chang 2020	11	40	14	40	3.7%	0.79 [0.41, 1.52]
**Subtotal (95% CI)**		**40**		**40**	**3.7%**	**0.79 [0.41, 1.52]**
Total events	11		14			
Heterogeneity: Not applicable						
Test for overall effect: Z = 0.72 (P = 0.47)						
**1.12.3 Open-label**						
Fujii 2020	30	105	25	102	5.7%	1.17 [0.74, 1.84]
Mohamed 2020	26	45	23	43	6.8%	1.08 [0.74, 1.57]
Raghu 2021	3	120	31	120	1.5%	0.10 [0.03, 0.31]
Wani 2020	20	50	21	50	5.5%	0.95 [0.59, 1.52]
**Subtotal (95% CI)**		**320**		**315**	**19.6%**	**0.72 [0.37, 1.40]**
Total events	79		100			
Heterogeneity: Tau² = 0.36; Chi² = 19.33, df = 3 (P = 0.0002); I² = 84%						
Test for overall effect: Z = 0.96 (P = 0.34)						
**1.12.4 Unclear**						
Hussein 2021	17	47	21	47	5.2%	0.81 [0.49, 1.33]
Jamshidi 2021	3	29	11	29	1.5%	0.27 [0.08, 0.88]
Lv 2020	15	61	24	56	4.8%	0.57 [0.34, 0.98]
Ranie AP 2022	4	40	4	40	1.2%	1.00 [0.27, 3.72]
**Subtotal (95% CI)**		**177**		**172**	**12.8%**	**0.65 [0.44, 0.95]**
Total events	39		60			
Heterogeneity: Tau² = 0.03; Chi² = 3.56, df = 3 (P = 0.31); I² = 16%						
Test for overall effect: Z = 2.22 (P = 0.03)						
**Total (95% CI)**		**1820**		**1815**	**100.0%**	**0.86 [0.74, 1.01]**
Total events	599		653			
Heterogeneity: Tau² = 0.05; Chi² = 43.15, df = 21 (P = 0.003); I² = 51%						
Test for overall effect: Z = 1.88 (P = 0.06)						
Test for subgroup differences: Chi² = 3.97, df = 3 (P = 0.26), I² = 24.4%						

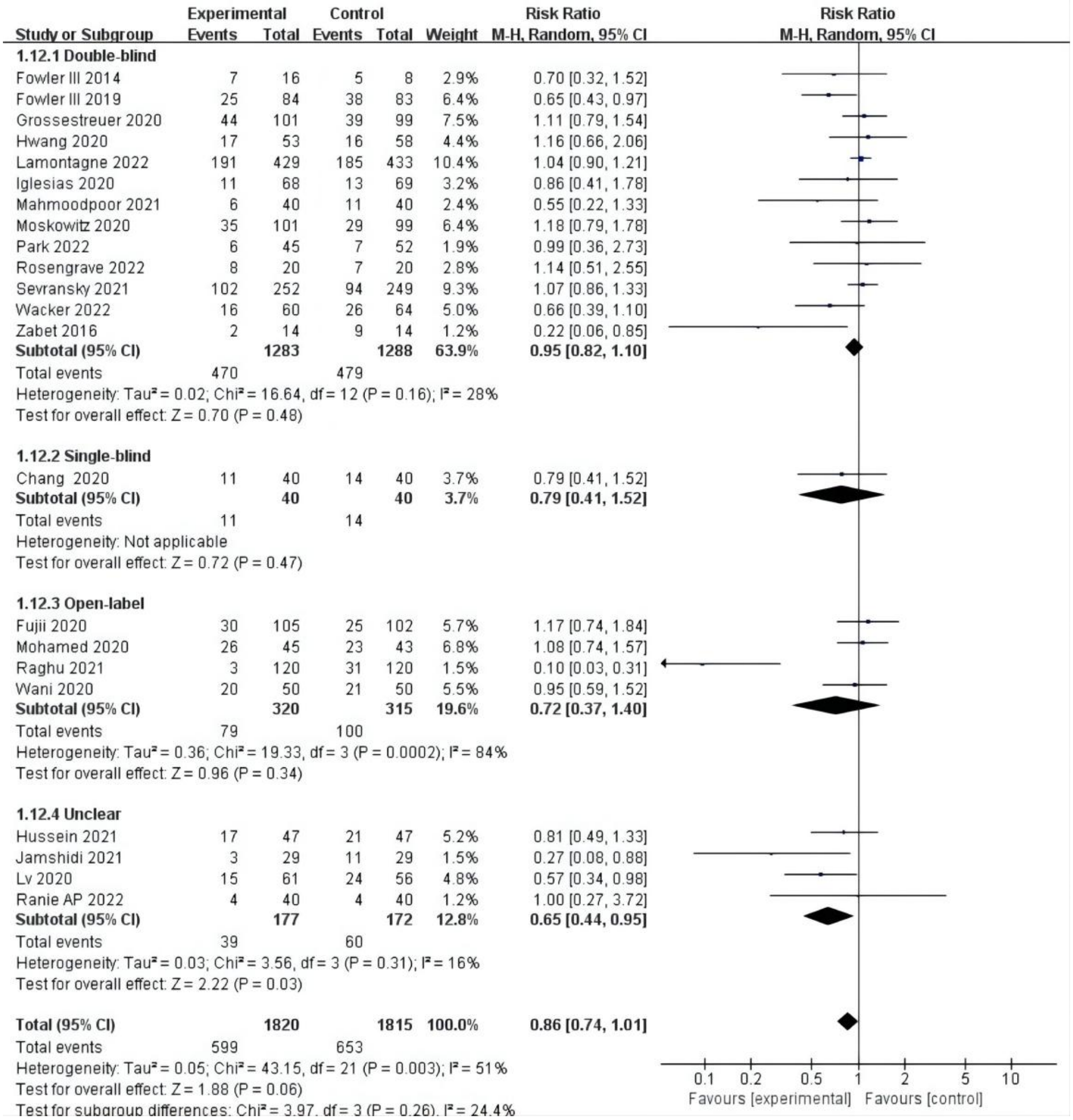

C

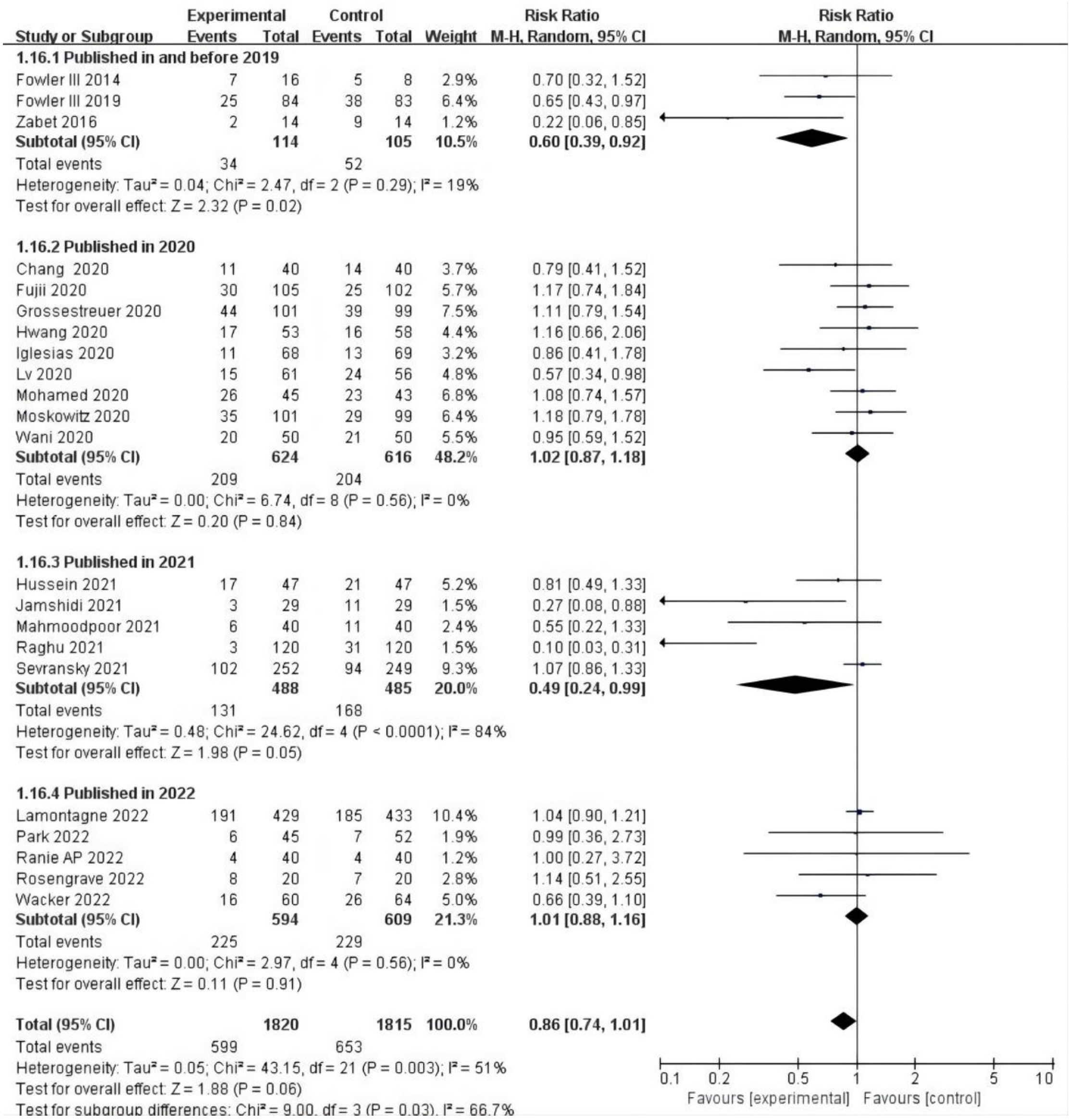

D

Supplementary figure.2. Forest plots for subgroup analysis of overall mortality according to protocol (A),the quality of the studies(B),blind methord(C)and publication year(D).
