# Supplementary material for: IV Vitamin C in Sepsis: A Latest Systematic Review and Meta-Analysis
Source: Int J Clin Pract. 2023 Jan 24;2023:6733465. doi: 10.1155/2023/6733465 (PMC9889164; doi:10.1155/2023/6733465)
Supplement: Supplementary Materials — Supplementary Figure 1: Forest plots for the vitamin C effect on the overall mortality after removing the study of Raghu et al. Supplementary Figure 2: Forest plots for subgroup analysis of overall mortality according to protocol (A), the quality of the studies (B), blind method (C), and publication year (D). Supplementary Figure 3: Funnel plot for publication bias, which compared the 28-day mortality in the vitamin C group and control group. Supplementary Figure 4: Funnel plot for publication bias, which compared the hospital mortality in the vitamin C group and control group. Supplementary Figure 5: Forest plots for the vitamin C effect on the hospital mortality after removing the study of Raghu et al. Supplementary Figure 6: Funnel plot for publication bias, which compared the vasopressors duration in the vitamin C group and control group. Supplementary Figure 7: Sensitivity analysis for vasopressors duration. Supplementary Figure 8: Sensitivity analysis for duration of mechanical ventilation. After removal of the study of Mahmoodpoor et al. [17], the heterogeneity of the included studies significantly decreased, and the result was also changed (E). Supplementary Figure 9: Funnel plot for publication bias, which compared the length of ICU stay in the vitamin C group and control group. Supplementary Figure 10: Funnel plot for publication bias which compared the hospital stay in the vitamin C group and control group. Supplementary Figure 11: Sensitivity analysis for length of hospital stay. After removal of the study of Raghu et al., the heterogeneity changed significantly, but the result did not change (F). Supplementary Figure 12: Forest plots for the vitamin C effect on the overall mortality after removing the study of Jamshidi et al., Raghu et al., and Zabet et al. [file 6733465.f1.zip › supplementary figure 11.pdf]

	Experimental			Control				Std. Mean Difference
**Study or Subgroup**	**Mean**	**SD**	**Total**	**Mean**	**SD**	**Total**	**Weight**	**IV, Random, 95% CI**
Fujii 2020	14.83	14.67	107	14.87	14.74	104	0.0%	-0.00 [-0.27, 0.27]
Hussein 2021	9.447	4.226	47	11.17	5.036	47	9.6%	-0.37 [-0.78, 0.04]
Hwang 2020	15.33	7.41	53	16.17	12.59	58	10.6%	-0.08 [-0.45, 0.29]
Iglesias 2020	11.5	6.8	68	11	6.2	69	11.7%	0.08 [-0.26, 0.41]
Mohamed 2020	31.58	31.06	45	20.9	15.01	52	9.7%	0.44 [0.04, 0.85]
Raghu 2021	13.64	9.04	120	10.28	5.93	120	14.4%	0.44 [0.18, 0.69]
Rosengrave 2022	21.6	18.1	20	28.48	31.67	20	5.6%	-0.26 [-0.88, 0.36]
Sevransky 2021	11	8.15	252	10.33	8.89	249	17.4%	0.08 [-0.10, 0.25]
Wacker 2022	10.97	11.85	60	7.53	6.44	64	11.1%	0.36 [0.01, 0.72]
Wani 2020	11.82	7.36	50	10.7	6.39	50	10.0%	0.16 [-0.23, 0.55]
**Total (95% CI)**			**715**			**729**	**100.0%**	**0.13 [-0.04, 0.30]**

Heterogeneity: Tau² = 0.04; Chi² = 18.39, df = 8 (P = 0.02); I² = 57%

Test for overall effect: Z = 1.45 (P = 0.15)

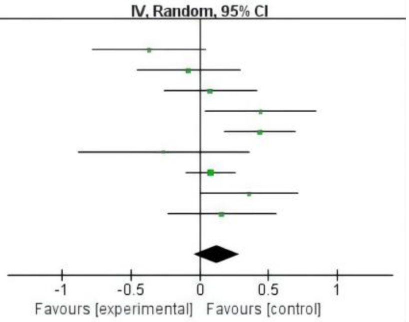

A

	Experimental			Control				Std. Mean Difference
**Study or Subgroup**	**Mean**	**SD**	**Total**	**Mean**	**SD**	**Total**	**Weight**	**IV, Random, 95% CI**
Fujii 2020	14.83	14.67	107	14.87	14.74	104	13.9%	-0.00 [-0.27, 0.27]
Hussein 2021	9.447	4.226	47	11.17	5.036	47	0.0%	-0.37 [-0.78, 0.04]
Hwang 2020	15.33	7.41	53	16.17	12.59	58	9.4%	-0.08 [-0.45, 0.29]
Iglesias 2020	11.5	6.8	68	11	6.2	69	10.8%	0.08 [-0.26, 0.41]
Mohamed 2020	31.58	31.06	45	20.9	15.01	52	8.4%	0.44 [0.04, 0.85]
Raghu 2021	13.64	9.04	120	10.28	5.93	120	14.6%	0.44 [0.18, 0.69]
Rosengrave 2022	21.6	18.1	20	28.48	31.67	20	4.2%	-0.26 [-0.88, 0.36]
Sevransky 2021	11	8.15	252	10.33	8.89	249	19.9%	0.08 [-0.10, 0.25]
Wacker 2022	10.97	11.85	60	7.53	6.44	64	10.0%	0.36 [0.01, 0.72]
Wani 2020	11.82	7.36	50	10.7	6.39	50	8.7%	0.16 [-0.23, 0.55]
**Total (95% CI)**			**775**			**786**	**100.0%**	**0.16 [0.02, 0.30]**

Heterogeneity: Tau² = 0.02; Chi² = 13.47, df = 8 (P = 0.10); I² = 41%

Test for overall effect: Z = 2.22 (P = 0.03)

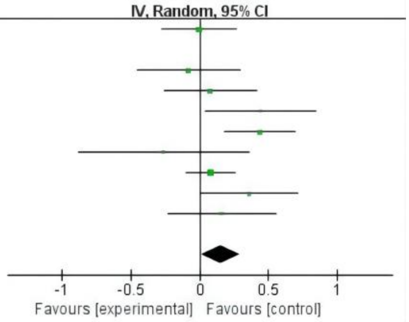

B

	Experimental			Control				Std. Mean Difference
**Study or Subgroup**	**Mean**	**SD**	**Total**	**Mean**	**SD**	**Total**	**Weight**	**IV, Random, 95% CI**
Fujii 2020	14.83	14.67	107	14.87	14.74	104	13.5%	-0.00 [-0.27, 0.27]
Hussein 2021	9.447	4.226	47	11.17	5.036	47	9.2%	-0.37 [-0.78, 0.04]
Hwang 2020	15.33	7.41	53	16.17	12.59	58	0.0%	-0.08 [-0.45, 0.29]
Iglesias 2020	11.5	6.8	68	11	6.2	69	11.3%	0.08 [-0.26, 0.41]
Mohamed 2020	31.58	31.06	45	20.9	15.01	52	9.3%	0.44 [0.04, 0.85]
Raghu 2021	13.64	9.04	120	10.28	5.93	120	14.1%	0.44 [0.18, 0.69]
Rosengrave 2022	21.6	18.1	20	28.48	31.67	20	5.2%	-0.26 [-0.88, 0.36]
Sevransky 2021	11	8.15	252	10.33	8.89	249	17.2%	0.08 [-0.10, 0.25]
Wacker 2022	10.97	11.85	60	7.53	6.44	64	10.7%	0.36 [0.01, 0.72]
Wani 2020	11.82	7.36	50	10.7	6.39	50	9.6%	0.16 [-0.23, 0.55]
**Total (95% CI)**			**769**			**775**	**100.0%**	**0.13 [-0.03, 0.29]**

Heterogeneity: Tau² = 0.03; Chi² = 18.13, df = 8 (P = 0.02); I² = 56%

Test for overall effect: Z = 1.58 (P = 0.11)

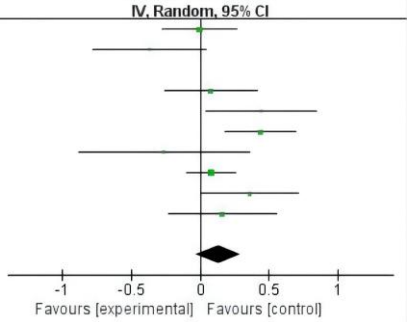

C

	Experimental			Control				Std. Mean Difference
**Study or Subgroup**	**Mean**	**SD**	**Total**	**Mean**	**SD**	**Total**	**Weight**	**IV, Random, 95% CI**
Fujii 2020	14.83	14.67	107	14.87	14.74	104	13.6%	-0.00 [-0.27, 0.27]
Hussein 2021	9.447	4.226	47	11.17	5.036	47	9.4%	-0.37 [-0.78, 0.04]
Hwang 2020	15.33	7.41	53	16.17	12.59	58	10.4%	-0.08 [-0.45, 0.29]
Iglesias 2020	11.5	6.8	68	11	6.2	69	0.0%	0.08 [-0.26, 0.41]
Mohamed 2020	31.58	31.06	45	20.9	15.01	52	9.5%	0.44 [0.04, 0.85]
Raghu 2021	13.64	9.04	120	10.28	5.93	120	14.0%	0.44 [0.18, 0.69]
Rosengrave 2022	21.6	18.1	20	28.48	31.67	20	5.5%	-0.26 [-0.88, 0.36]
Sevransky 2021	11	8.15	252	10.33	8.89	249	16.9%	0.08 [-0.10, 0.25]
Wacker 2022	10.97	11.85	60	7.53	6.44	64	10.9%	0.36 [0.01, 0.72]
Wani 2020	11.82	7.36	50	10.7	6.39	50	9.8%	0.16 [-0.23, 0.55]
**Total (95% CI)**			**754**			**764**	**100.0%**	**0.11 [-0.06, 0.28]**

Heterogeneity: Tau² = 0.04; Chi² = 19.26, df = 8 (P = 0.01); I² = 58%

Test for overall effect: Z = 1.32 (P = 0.19)

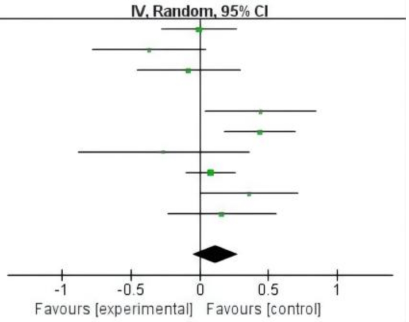

D

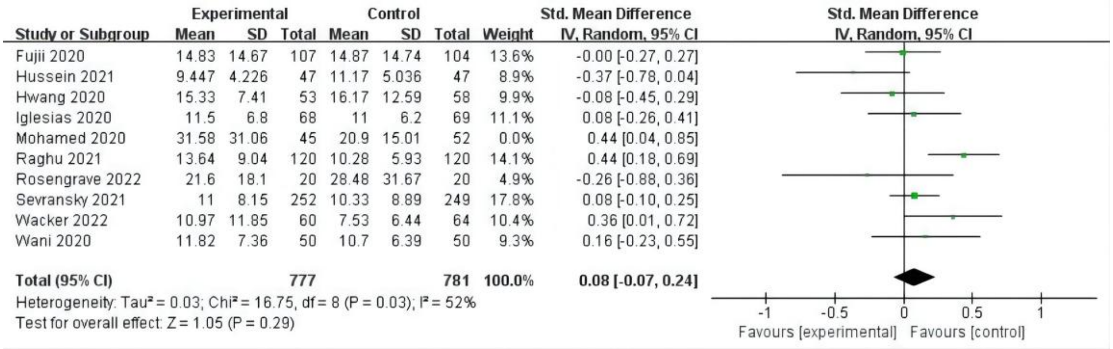

Study or Subgroup	Experimental Mean	Experimental SD	Experimental Total	Control Mean	Control SD	Control Total	Weight	Std. Mean Difference IV, Random, 95% CI
Fujii 2020	14.83	14.67	107	14.87	14.74	104	13.6%	-0.00 [-0.27, 0.27]
Hussein 2021	9.447	4.226	47	11.17	5.036	47	8.9%	-0.37 [-0.78, 0.04]
Hwang 2020	15.33	7.41	53	16.17	12.59	58	9.9%	-0.08 [-0.45, 0.29]
Iglesias 2020	11.5	6.8	68	11	6.2	69	11.1%	0.08 [-0.26, 0.41]
Mohamed 2020	31.58	31.06	45	20.9	15.01	52	0.0%	0.44 [0.04, 0.85]
Raghu 2021	13.64	9.04	120	10.28	5.93	120	14.1%	0.44 [0.18, 0.69]
Rosengrave 2022	21.6	18.1	20	28.48	31.67	20	4.9%	-0.26 [-0.88, 0.36]
Sevransky 2021	11	8.15	252	10.33	8.89	249	17.8%	0.08 [-0.10, 0.25]
Wacker 2022	10.97	11.85	60	7.53	6.44	64	10.4%	0.36 [0.01, 0.72]
Wani 2020	11.82	7.36	50	10.7	6.39	50	9.3%	0.16 [-0.23, 0.55]
**Total (95% CI)**			**777**			**781**	**100.0%**	**0.08 [-0.07, 0.24]**

Heterogeneity: Tau² = 0.03; Chi² = 16.75, df = 8 (P = 0.03); I² = 52%
Test for overall effect: Z = 1.05 (P = 0.29)

E

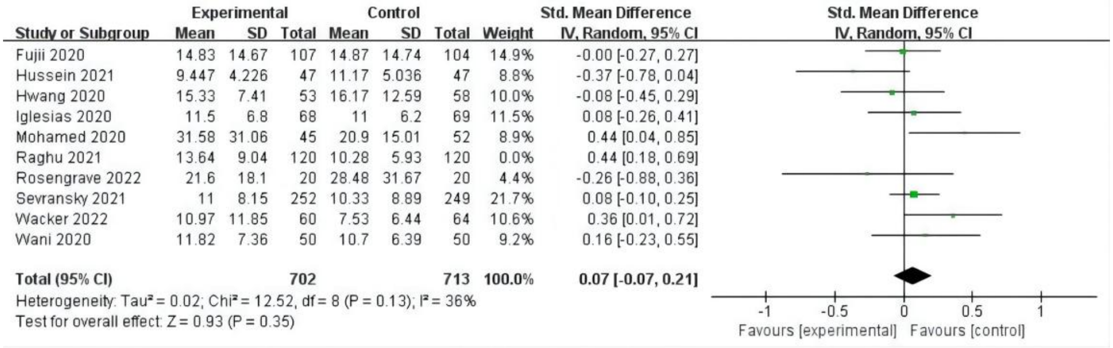

Study or Subgroup	Experimental Mean	Experimental SD	Experimental Total	Control Mean	Control SD	Control Total	Weight	Std. Mean Difference IV, Random, 95% CI
Fujii 2020	14.83	14.67	107	14.87	14.74	104	14.9%	-0.00 [-0.27, 0.27]
Hussein 2021	9.447	4.226	47	11.17	5.036	47	8.8%	-0.37 [-0.78, 0.04]
Hwang 2020	15.33	7.41	53	16.17	12.59	58	10.0%	-0.08 [-0.45, 0.29]
Iglesias 2020	11.5	6.8	68	11	6.2	69	11.5%	0.08 [-0.26, 0.41]
Mohamed 2020	31.58	31.06	45	20.9	15.01	52	8.9%	0.44 [0.04, 0.85]
Raghu 2021	13.64	9.04	120	10.28	5.93	120	0.0%	0.44 [0.18, 0.69]
Rosengrave 2022	21.6	18.1	20	28.48	31.67	20	4.4%	-0.26 [-0.88, 0.36]
Sevransky 2021	11	8.15	252	10.33	8.89	249	21.7%	0.08 [-0.10, 0.25]
Wacker 2022	10.97	11.85	60	7.53	6.44	64	10.6%	0.36 [0.01, 0.72]
Wani 2020	11.82	7.36	50	10.7	6.39	50	9.2%	0.16 [-0.23, 0.55]
**Total (95% CI)**			**702**			**713**	**100.0%**	**0.07 [-0.07, 0.21]**

Heterogeneity: Tau² = 0.02; Chi² = 12.52, df = 8 (P = 0.13); I² = 36%
Test for overall effect: Z = 0.93 (P = 0.35)

F

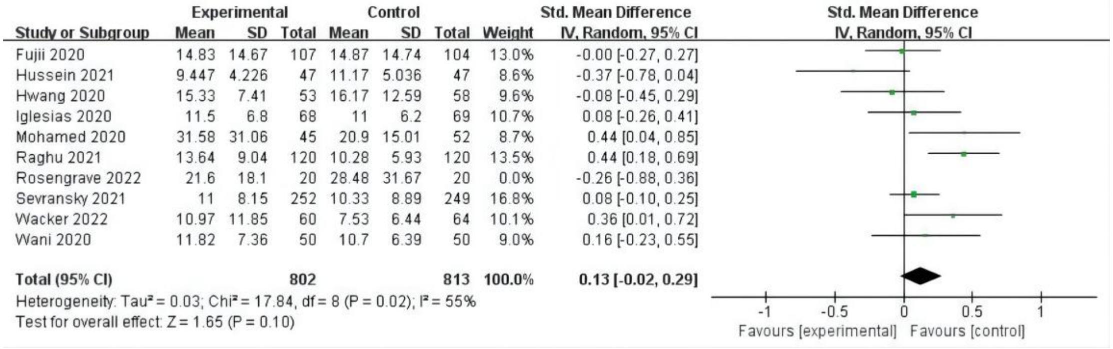

Study or Subgroup	Experimental Mean	Experimental SD	Experimental Total	Control Mean	Control SD	Control Total	Weight	Std. Mean Difference IV, Random, 95% CI
Fujii 2020	14.83	14.67	107	14.87	14.74	104	13.0%	-0.00 [-0.27, 0.27]
Hussein 2021	9.447	4.226	47	11.17	5.036	47	8.6%	-0.37 [-0.78, 0.04]
Hwang 2020	15.33	7.41	53	16.17	12.59	58	9.6%	-0.08 [-0.45, 0.29]
Iglesias 2020	11.5	6.8	68	11	6.2	69	10.7%	0.08 [-0.26, 0.41]
Mohamed 2020	31.58	31.06	45	20.9	15.01	52	8.7%	0.44 [0.04, 0.85]
Raghu 2021	13.64	9.04	120	10.28	5.93	120	13.5%	0.44 [0.18, 0.69]
Rosengrave 2022	21.6	18.1	20	28.48	31.67	20	0.0%	-0.26 [-0.88, 0.36]
Sevransky 2021	11	8.15	252	10.33	8.89	249	16.8%	0.08 [-0.10, 0.25]
Wacker 2022	10.97	11.85	60	7.53	6.44	64	10.1%	0.36 [0.01, 0.72]
Wani 2020	11.82	7.36	50	10.7	6.39	50	9.0%	0.16 [-0.23, 0.55]
**Total (95% CI)**			**802**			**813**	**100.0%**	**0.13 [-0.02, 0.29]**

Heterogeneity: Tau² = 0.03; Chi² = 17.84, df = 8 (P = 0.02); I² = 55%
Test for overall effect: Z = 1.65 (P = 0.10)

G

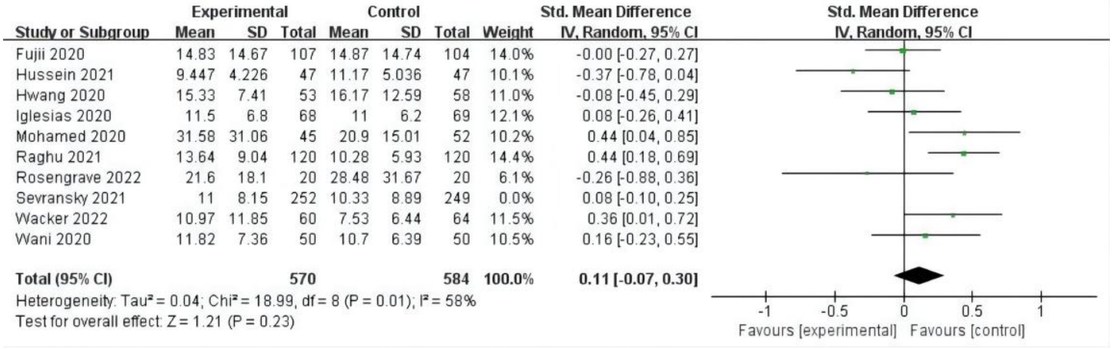

Study or Subgroup	Experimental Mean	Experimental SD	Experimental Total	Control Mean	Control SD	Control Total	Weight	Std. Mean Difference IV, Random, 95% CI
Fujii 2020	14.83	14.67	107	14.87	14.74	104	14.0%	-0.00 [-0.27, 0.27]
Hussein 2021	9.447	4.226	47	11.17	5.036	47	10.1%	-0.37 [-0.78, 0.04]
Hwang 2020	15.33	7.41	53	16.17	12.59	58	11.0%	-0.08 [-0.45, 0.29]
Iglesias 2020	11.5	6.8	68	11	6.2	69	12.1%	0.08 [-0.26, 0.41]
Mohamed 2020	31.58	31.06	45	20.9	15.01	52	10.2%	0.44 [0.04, 0.85]
Raghu 2021	13.64	9.04	120	10.28	5.93	120	14.4%	0.44 [0.18, 0.69]
Rosengrave 2022	21.6	18.1	20	28.48	31.67	20	6.1%	-0.26 [-0.88, 0.36]
Sevransky 2021	11	8.15	252	10.33	8.89	249	0.0%	0.08 [-0.10, 0.25]
Wacker 2022	10.97	11.85	60	7.53	6.44	64	11.5%	0.36 [0.01, 0.72]
Wani 2020	11.82	7.36	50	10.7	6.39	50	10.5%	0.16 [-0.23, 0.55]
**Total (95% CI)**			**570**			**584**	**100.0%**	**0.11 [-0.07, 0.30]**

Heterogeneity: Tau² = 0.04; Chi² = 18.99, df = 8 (P = 0.01); I² = 58%
Test for overall effect: Z = 1.21 (P = 0.23)

H

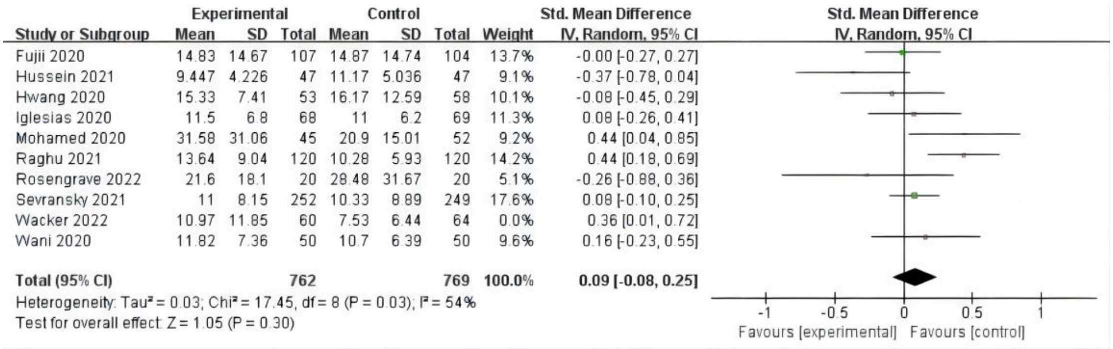

I

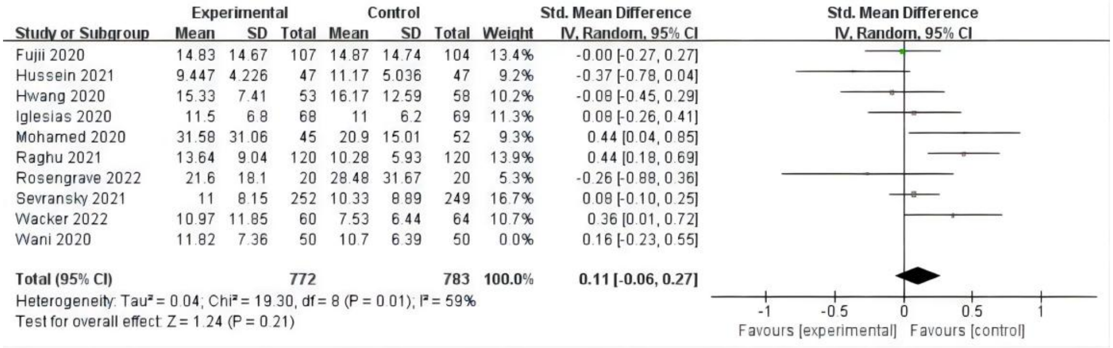

J

Supplementary figure 11. Sensitivity analysis for length of hospital stay.After removal of the study of Raghu et al. the heterogeneity changed significantly, but the result didn’t change(F).
