# Supplementary material for: IV Vitamin C in Sepsis: A Latest Systematic Review and Meta-Analysis
Source: Int J Clin Pract. 2023 Jan 24;2023:6733465. doi: 10.1155/2023/6733465 (PMC9889164; doi:10.1155/2023/6733465)
Supplement: Supplementary Materials — Supplementary Figure 1: Forest plots for the vitamin C effect on the overall mortality after removing the study of Raghu et al. Supplementary Figure 2: Forest plots for subgroup analysis of overall mortality according to protocol (A), the quality of the studies (B), blind method (C), and publication year (D). Supplementary Figure 3: Funnel plot for publication bias, which compared the 28-day mortality in the vitamin C group and control group. Supplementary Figure 4: Funnel plot for publication bias, which compared the hospital mortality in the vitamin C group and control group. Supplementary Figure 5: Forest plots for the vitamin C effect on the hospital mortality after removing the study of Raghu et al. Supplementary Figure 6: Funnel plot for publication bias, which compared the vasopressors duration in the vitamin C group and control group. Supplementary Figure 7: Sensitivity analysis for vasopressors duration. Supplementary Figure 8: Sensitivity analysis for duration of mechanical ventilation. After removal of the study of Mahmoodpoor et al. [17], the heterogeneity of the included studies significantly decreased, and the result was also changed (E). Supplementary Figure 9: Funnel plot for publication bias, which compared the length of ICU stay in the vitamin C group and control group. Supplementary Figure 10: Funnel plot for publication bias which compared the hospital stay in the vitamin C group and control group. Supplementary Figure 11: Sensitivity analysis for length of hospital stay. After removal of the study of Raghu et al., the heterogeneity changed significantly, but the result did not change (F). Supplementary Figure 12: Forest plots for the vitamin C effect on the overall mortality after removing the study of Jamshidi et al., Raghu et al., and Zabet et al. [file 6733465.f1.zip › supplementary figure 5.pdf]

Study or Subgroup	Experimental Events	Experimental Total	Control Events	Control Total	Weight	Risk Ratio M-H, Random, 95% CI
Fujii 2020	25	107	21	103	20.5%	1.15 [0.69, 1.91]
Hwang 2020	13	53	11	58	10.9%	1.29 [0.63, 2.63]
Jamshidi 2021	3	29	11	29	4.1%	0.27 [0.08, 0.88]
Iglesias 2020	11	68	13	69	10.4%	0.86 [0.41, 1.78]
Mahmoodpoor 2021	6	40	11	40	7.0%	0.55 [0.22, 1.33]
Moskowitz 2020	28	101	23	99	23.6%	1.19 [0.74, 1.92]
Raghu 2021	3	120	31	120	0.0%	0.10 [0.03, 0.31]
Ranie AP 2022	4	40	4	40	3.2%	1.00 [0.27, 3.72]
Rosengrave 2022	7	20	7	20	7.8%	1.00 [0.43, 2.33]
Wani 2020	12	50	14	50	12.5%	0.86 [0.44, 1.66]
**Total (95% CI)**		**508**		**508**	**100.0%**	**0.97 [0.76, 1.23]**
Total events	109		115			

Heterogeneity: Tau² = 0.00; Chi² = 8.18, df = 8 (P = 0.42); I² = 2%
Test for overall effect: Z = 0.27 (P = 0.79)

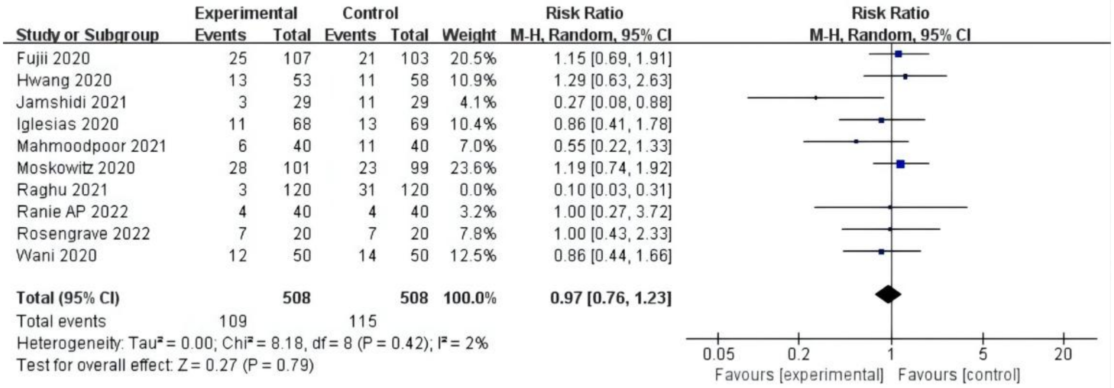

supplementary figure 5. Forest plots for the vitamin C effect on the hospital mortality after removing the study of Raghu et al.
