# Supplementary material for: IV Vitamin C in Sepsis: A Latest Systematic Review and Meta-Analysis
Source: Int J Clin Pract. 2023 Jan 24;2023:6733465. doi: 10.1155/2023/6733465 (PMC9889164; doi:10.1155/2023/6733465)
Supplement: Supplementary Materials — Supplementary Figure 1: Forest plots for the vitamin C effect on the overall mortality after removing the study of Raghu et al. Supplementary Figure 2: Forest plots for subgroup analysis of overall mortality according to protocol (A), the quality of the studies (B), blind method (C), and publication year (D). Supplementary Figure 3: Funnel plot for publication bias, which compared the 28-day mortality in the vitamin C group and control group. Supplementary Figure 4: Funnel plot for publication bias, which compared the hospital mortality in the vitamin C group and control group. Supplementary Figure 5: Forest plots for the vitamin C effect on the hospital mortality after removing the study of Raghu et al. Supplementary Figure 6: Funnel plot for publication bias, which compared the vasopressors duration in the vitamin C group and control group. Supplementary Figure 7: Sensitivity analysis for vasopressors duration. Supplementary Figure 8: Sensitivity analysis for duration of mechanical ventilation. After removal of the study of Mahmoodpoor et al. [17], the heterogeneity of the included studies significantly decreased, and the result was also changed (E). Supplementary Figure 9: Funnel plot for publication bias, which compared the length of ICU stay in the vitamin C group and control group. Supplementary Figure 10: Funnel plot for publication bias which compared the hospital stay in the vitamin C group and control group. Supplementary Figure 11: Sensitivity analysis for length of hospital stay. After removal of the study of Raghu et al., the heterogeneity changed significantly, but the result did not change (F). Supplementary Figure 12: Forest plots for the vitamin C effect on the overall mortality after removing the study of Jamshidi et al., Raghu et al., and Zabet et al. [file 6733465.f1.zip › supplementary figure 8.pdf]

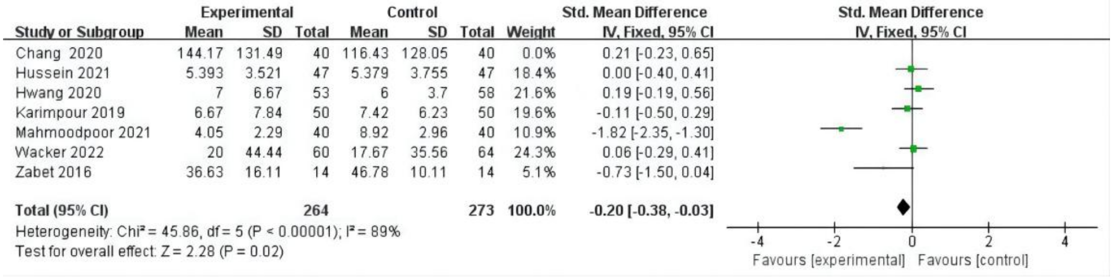
Experimental
Control
Std. Mean Difference
Std. Mean Difference
Study or Subgroup Mean SD Total Mean SD Total Weight IV, Fixed, 95% CI
IV, Fixed, 95% CI
Chang 2020 144.17 131.49 40 116.43 128.05 40 0.0% 0.21 [-0.23, 0.65]
Hussein 2021 5.393 3.521 47 5.379 3.755 47 18.4% 0.00 [-0.40, 0.41]
Hwang 2020 7 6.67 53 6 3.7 58 21.6% 0.19 [-0.19, 0.56]
Karimpour 2019 6.67 7.84 50 7.42 6.23 50 19.6% -0.11 [-0.50, 0.29]
Mahmoodpoor 2021 4.05 2.29 40 8.92 2.96 40 10.9% -1.82 [-2.35, -1.30]
Wacker 2022 20 44.44 60 17.67 35.56 64 24.3% 0.06 [-0.29, 0.41]
Zabet 2016 36.63 16.11 14 46.78 10.11 14 5.1% -0.73 [-1.50, 0.04]
Total (95% CI) 264 273 100.0% -0.20 [-0.38, -0.03]
Heterogeneity: Chi² = 45.86, df = 5 (P < 0.00001); I² = 89%
Test for overall effect: Z = 2.28 (P = 0.02)
-4 -2 0 2 4
Favours [experimental] Favours [control]

A

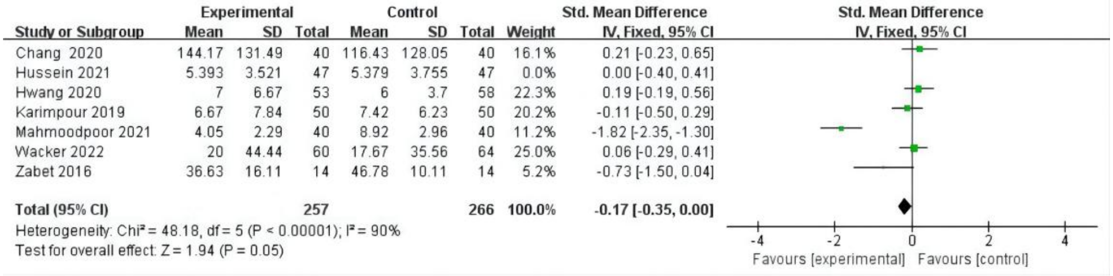
Experimental
Control
Std. Mean Difference
Std. Mean Difference
Study or Subgroup Mean SD Total Mean SD Total Weight IV, Fixed, 95% CI
IV, Fixed, 95% CI
Chang 2020 144.17 131.49 40 116.43 128.05 40 16.1% 0.21 [-0.23, 0.65]
Hussein 2021 5.393 3.521 47 5.379 3.755 47 0.0% 0.00 [-0.40, 0.41]
Hwang 2020 7 6.67 53 6 3.7 58 22.3% 0.19 [-0.19, 0.56]
Karimpour 2019 6.67 7.84 50 7.42 6.23 50 20.2% -0.11 [-0.50, 0.29]
Mahmoodpoor 2021 4.05 2.29 40 8.92 2.96 40 11.2% -1.82 [-2.35, -1.30]
Wacker 2022 20 44.44 60 17.67 35.56 64 25.0% 0.06 [-0.29, 0.41]
Zabet 2016 36.63 16.11 14 46.78 10.11 14 5.2% -0.73 [-1.50, 0.04]
Total (95% CI) 257 266 100.0% -0.17 [-0.35, 0.00]
Heterogeneity: Chi² = 48.18, df = 5 (P < 0.00001); I² = 90%
Test for overall effect: Z = 1.94 (P = 0.05)
-4 -2 0 2 4
Favours [experimental] Favours [control]

B

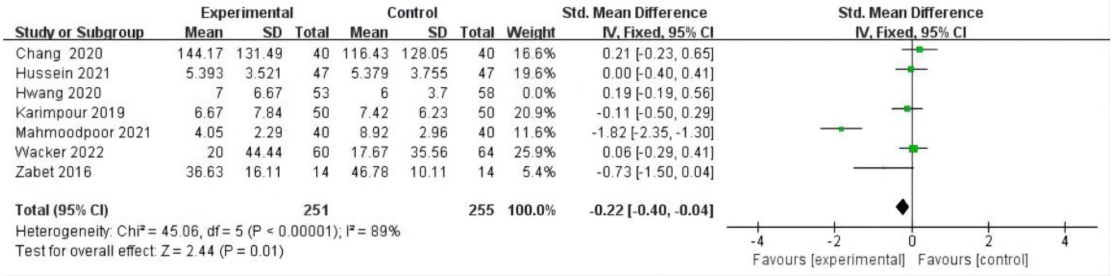
Experimental
Control
Std. Mean Difference
Std. Mean Difference
Study or Subgroup Mean SD Total Mean SD Total Weight IV, Fixed, 95% CI
IV, Fixed, 95% CI
Chang 2020 144.17 131.49 40 116.43 128.05 40 16.6% 0.21 [-0.23, 0.65]
Hussein 2021 5.393 3.521 47 5.379 3.755 47 19.6% 0.00 [-0.40, 0.41]
Hwang 2020 7 6.67 53 6 3.7 58 0.0% 0.19 [-0.19, 0.56]
Karimpour 2019 6.67 7.84 50 7.42 6.23 50 20.9% -0.11 [-0.50, 0.29]
Mahmoodpoor 2021 4.05 2.29 40 8.92 2.96 40 11.6% -1.82 [-2.35, -1.30]
Wacker 2022 20 44.44 60 17.67 35.56 64 25.9% 0.06 [-0.29, 0.41]
Zabet 2016 36.63 16.11 14 46.78 10.11 14 5.4% -0.73 [-1.50, 0.04]
Total (95% CI) 251 255 100.0% -0.22 [-0.40, -0.04]
Heterogeneity: Chi² = 45.06, df = 5 (P < 0.00001); I² = 89%
Test for overall effect: Z = 2.44 (P = 0.01)
-4 -2 0 2 4
Favours [experimental] Favours [control]

C

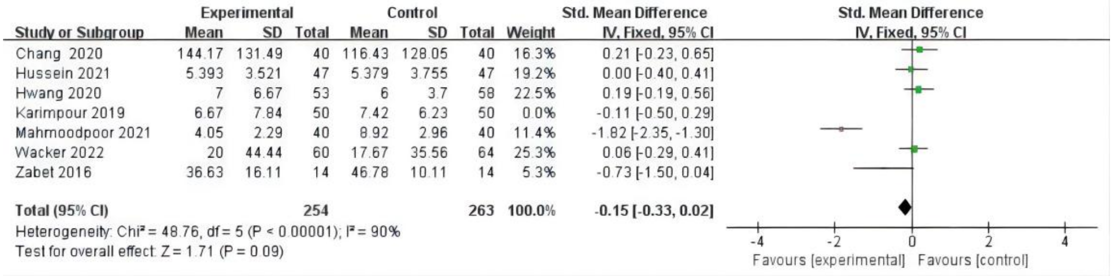
Experimental
Control
Std. Mean Difference
Std. Mean Difference
Study or Subgroup Mean SD Total Mean SD Total Weight IV, Fixed, 95% CI
IV, Fixed, 95% CI
Chang 2020 144.17 131.49 40 116.43 128.05 40 16.3% 0.21 [-0.23, 0.65]
Hussein 2021 5.393 3.521 47 5.379 3.755 47 19.2% 0.00 [-0.40, 0.41]
Hwang 2020 7 6.67 53 6 3.7 58 22.5% 0.19 [-0.19, 0.56]
Karimpour 2019 6.67 7.84 50 7.42 6.23 50 0.0% -0.11 [-0.50, 0.29]
Mahmoodpoor 2021 4.05 2.29 40 8.92 2.96 40 11.4% -1.82 [-2.35, -1.30]
Wacker 2022 20 44.44 60 17.67 35.56 64 25.3% 0.06 [-0.29, 0.41]
Zabet 2016 36.63 16.11 14 46.78 10.11 14 5.3% -0.73 [-1.50, 0.04]
Total (95% CI) 254 263 100.0% -0.15 [-0.33, 0.02]
Heterogeneity: Chi² = 48.76, df = 5 (P < 0.00001); I² = 90%
Test for overall effect: Z = 1.71 (P = 0.09)
-4 -2 0 2 4
Favours [experimental] Favours [control]

D

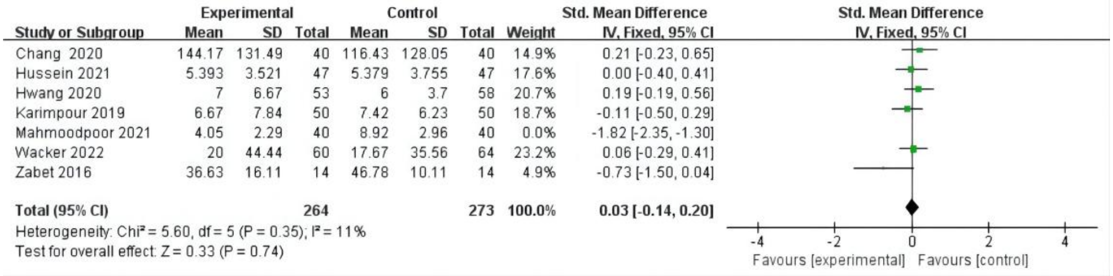
Experimental
Control
Std. Mean Difference
Std. Mean Difference
Study or Subgroup Mean SD Total Mean SD Total Weight IV, Fixed, 95% CI
IV, Fixed, 95% CI
Chang 2020 144.17 131.49 40 116.43 128.05 40 14.9% 0.21 [-0.23, 0.65]
Hussein 2021 5.393 3.521 47 5.379 3.755 47 17.6% 0.00 [-0.40, 0.41]
Hwang 2020 7 6.67 53 6 3.7 58 20.7% 0.19 [-0.19, 0.56]
Karimpour 2019 6.67 7.84 50 7.42 6.23 50 18.7% -0.11 [-0.50, 0.29]
Mahmoodpoor 2021 4.05 2.29 40 8.92 2.96 40 0.0% -1.82 [-2.35, -1.30]
Wacker 2022 20 44.44 60 17.67 35.56 64 23.2% 0.06 [-0.29, 0.41]
Zabet 2016 36.63 16.11 14 46.78 10.11 14 4.9% -0.73 [-1.50, 0.04]
Total (95% CI) 264 273 100.0% 0.03 [-0.14, 0.20]
Heterogeneity: Chi² = 5.60, df = 5 (P = 0.35); I² = 11%
Test for overall effect: Z = 0.33 (P = 0.74)
-4 -2 0 2 4
Favours [experimental] Favours [control]

E

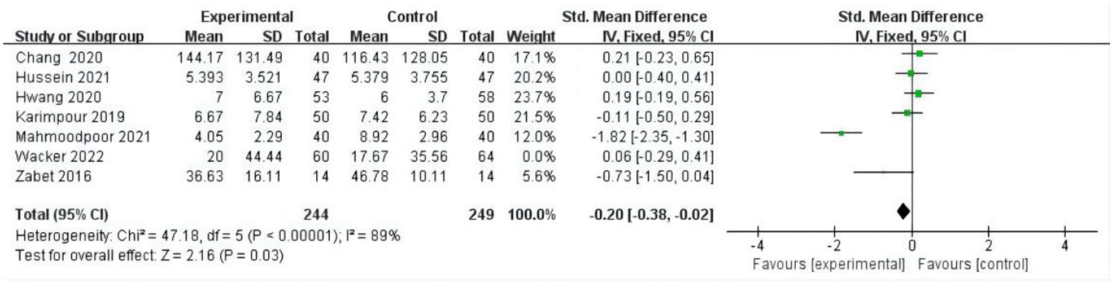

F

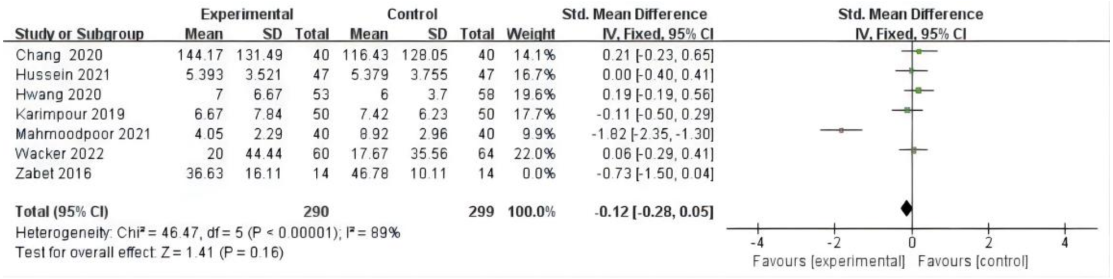

G

Supplementary figure 8. Sensitivity analysis for duration of mechanical ventilation.After removal of the study of Mahmoodpoor et al.[17], the heterogeneity of the included studies significantly decreased, and the result also was changed(E).
