# Supplementary material for: IV Vitamin C in Sepsis: A Latest Systematic Review and Meta-Analysis
Source: Int J Clin Pract. 2023 Jan 24;2023:6733465. doi: 10.1155/2023/6733465 (PMC9889164; doi:10.1155/2023/6733465)
Supplement: Supplementary Materials — Supplementary Figure 1: Forest plots for the vitamin C effect on the overall mortality after removing the study of Raghu et al. Supplementary Figure 2: Forest plots for subgroup analysis of overall mortality according to protocol (A), the quality of the studies (B), blind method (C), and publication year (D). Supplementary Figure 3: Funnel plot for publication bias, which compared the 28-day mortality in the vitamin C group and control group. Supplementary Figure 4: Funnel plot for publication bias, which compared the hospital mortality in the vitamin C group and control group. Supplementary Figure 5: Forest plots for the vitamin C effect on the hospital mortality after removing the study of Raghu et al. Supplementary Figure 6: Funnel plot for publication bias, which compared the vasopressors duration in the vitamin C group and control group. Supplementary Figure 7: Sensitivity analysis for vasopressors duration. Supplementary Figure 8: Sensitivity analysis for duration of mechanical ventilation. After removal of the study of Mahmoodpoor et al. [17], the heterogeneity of the included studies significantly decreased, and the result was also changed (E). Supplementary Figure 9: Funnel plot for publication bias, which compared the length of ICU stay in the vitamin C group and control group. Supplementary Figure 10: Funnel plot for publication bias which compared the hospital stay in the vitamin C group and control group. Supplementary Figure 11: Sensitivity analysis for length of hospital stay. After removal of the study of Raghu et al., the heterogeneity changed significantly, but the result did not change (F). Supplementary Figure 12: Forest plots for the vitamin C effect on the overall mortality after removing the study of Jamshidi et al., Raghu et al., and Zabet et al. [file 6733465.f1.zip › supplementay figure 12.pdf]

Study or Subgroup	Experimental Events	Experimental Total	Control Events	Control Total	Weight	Risk Ratio M-H, Random, 95% CI
Chang 2020	11	40	14	40	1.8%	0.79 [0.41, 1.52]
Fowler III 2014	7	16	5	8	1.3%	0.70 [0.32, 1.52]
Fowler III 2019	25	84	38	83	4.8%	0.65 [0.43, 0.97]
Fujii 2020	30	105	25	102	3.8%	1.17 [0.74, 1.84]
Grossestreuer 2020	44	101	39	99	7.2%	1.11 [0.79, 1.54]
Hussein 2021	17	47	21	47	3.2%	0.81 [0.49, 1.33]
Hwang 2020	17	53	16	58	2.4%	1.16 [0.66, 2.06]
Jamshidi 2021	3	29	11	29	0.0%	0.27 [0.08, 0.88]
Lamontagne 2022	191	429	185	433	34.2%	1.04 [0.90, 1.21]
Iglesias 2020	11	68	13	69	1.5%	0.86 [0.41, 1.78]
Lv 2020	15	61	24	56	2.8%	0.57 [0.34, 0.98]
Mahmoodpoor 2021	6	40	11	40	1.0%	0.55 [0.22, 1.33]
Mohamed 2020	26	45	23	43	5.6%	1.08 [0.74, 1.57]
Moskowitz 2020	35	101	29	99	4.8%	1.18 [0.79, 1.78]
Park 2022	6	45	7	52	0.8%	0.99 [0.36, 2.73]
Raghu 2021	3	120	31	120	0.0%	0.10 [0.03, 0.31]
Ranie AP 2022	4	40	4	40	0.5%	1.00 [0.27, 3.72]
Rosengrave 2022	8	20	7	20	1.2%	1.14 [0.51, 2.55]
Sevransky 2021	102	252	94	249	16.5%	1.07 [0.86, 1.33]
Wacker 2022	16	60	26	64	3.0%	0.66 [0.39, 1.10]
Wani 2020	20	50	21	50	3.6%	0.95 [0.59, 1.52]
Zabet 2016	2	14	9	14	0.0%	0.22 [0.06, 0.85]
**Total (95% CI)**		**1657**		**1652**	**100.0%**	**0.98 [0.90, 1.07]**
Total events	591		602			

Heterogeneity: Tau² = 0.00; Chi² = 17.66, df = 18 (P = 0.48); I² = 0%

Test for overall effect: Z = 0.41 (P = 0.68)

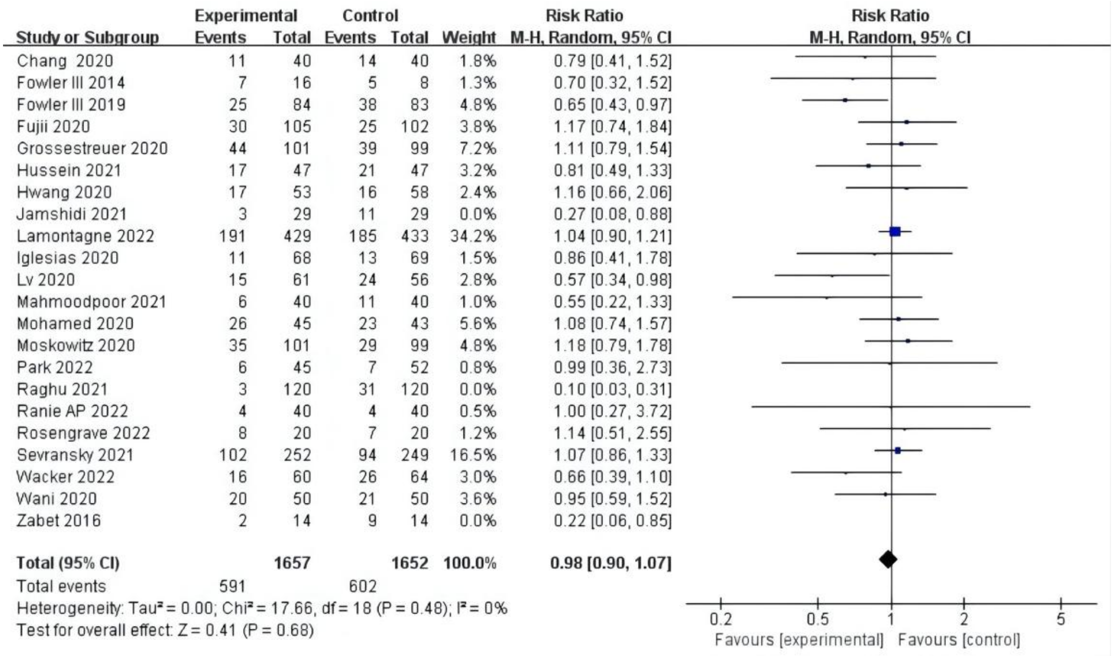

Supplementay figure 12. Forest plots for the vitamin C effect on the overall mortality.after removing the study of Jamshidi et al.Raghu et al.and Zabet et al.
