# Supplementary figures and images for: IV Vitamin C in Sepsis: A Latest Systematic Review and Meta-Analysis
Source: Int J Clin Pract. 2023 Jan 24;2023:6733465. doi: 10.1155/2023/6733465 (PMC9889164; doi:10.1155/2023/6733465)

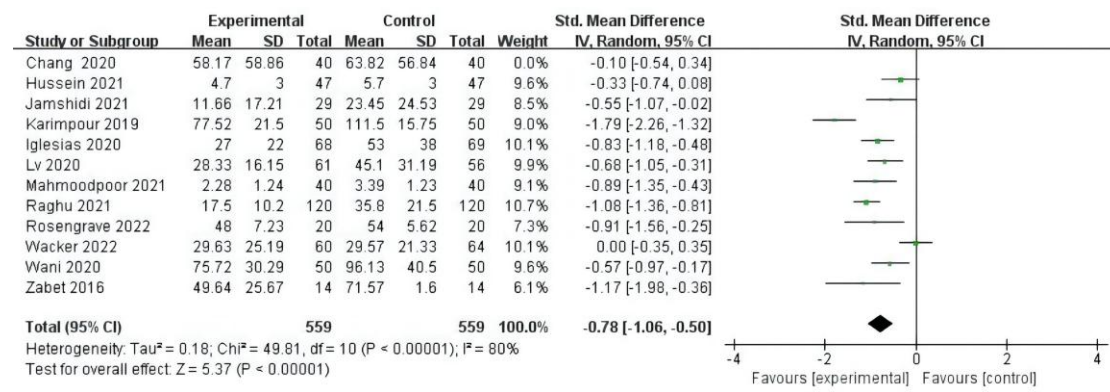

A

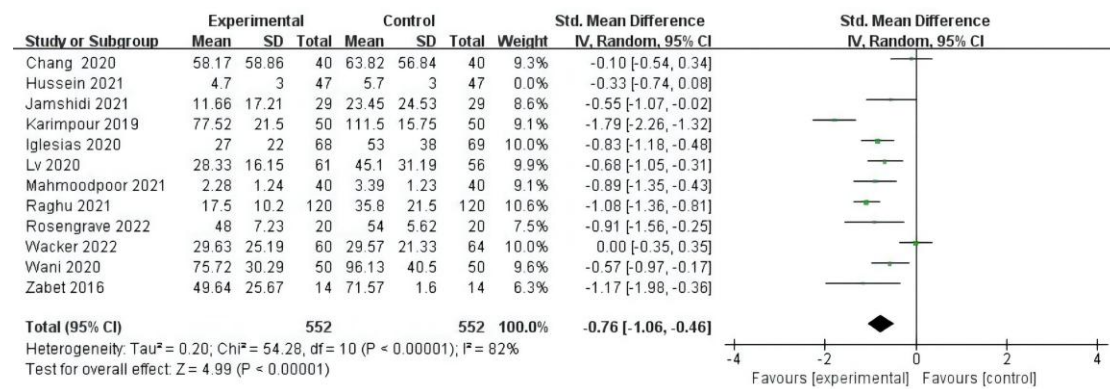

B

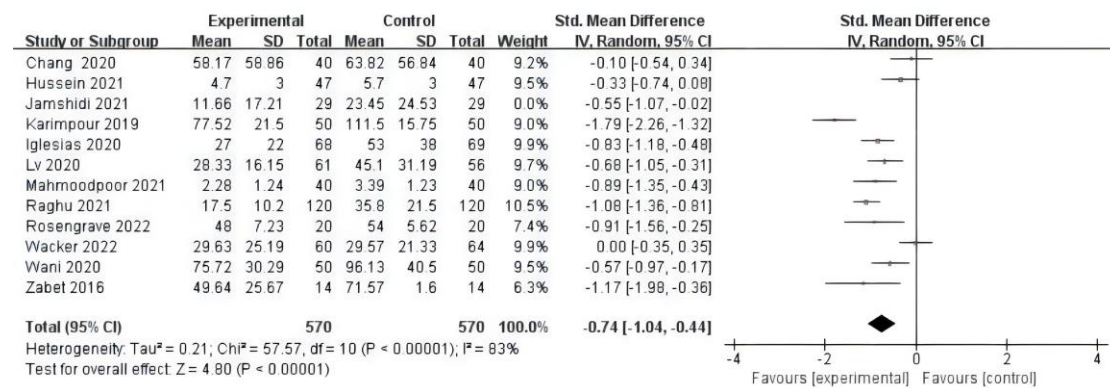

C

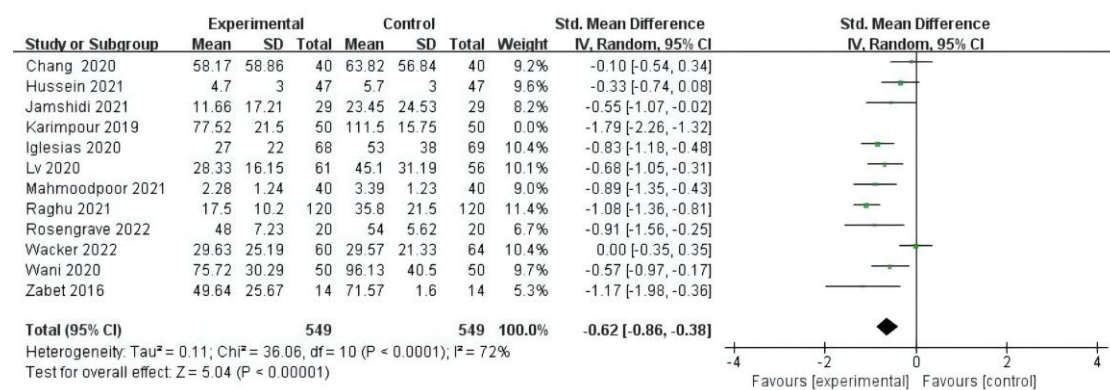

D

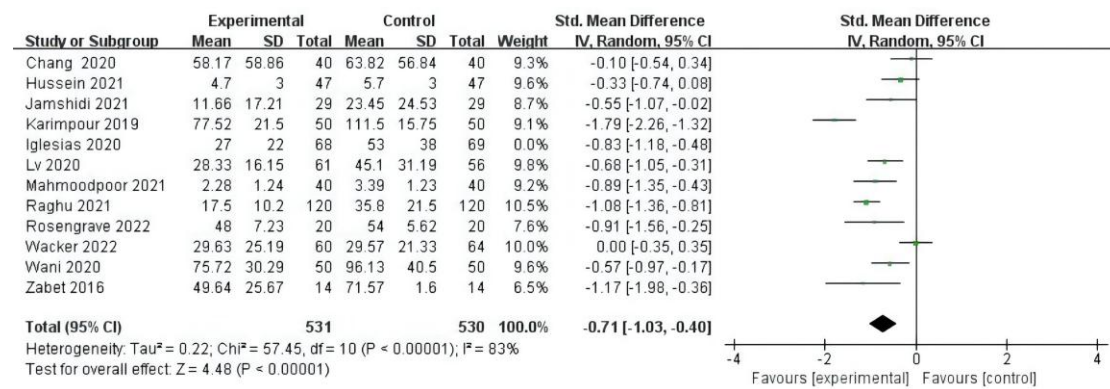

E

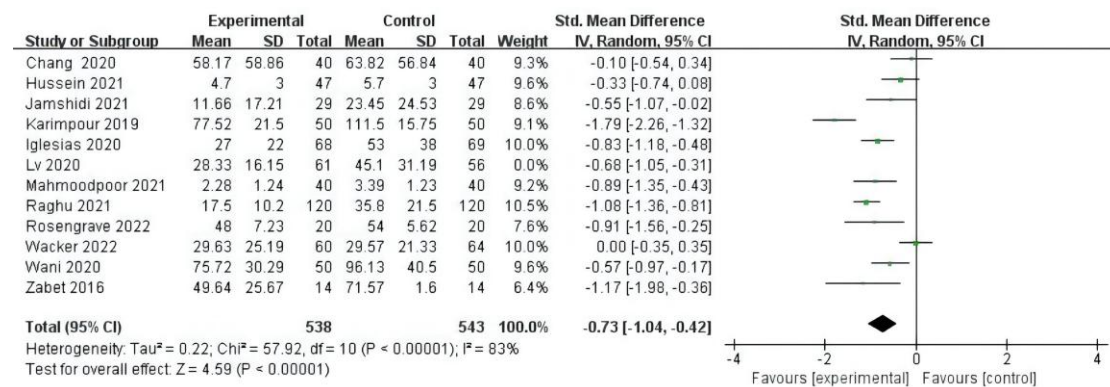

F

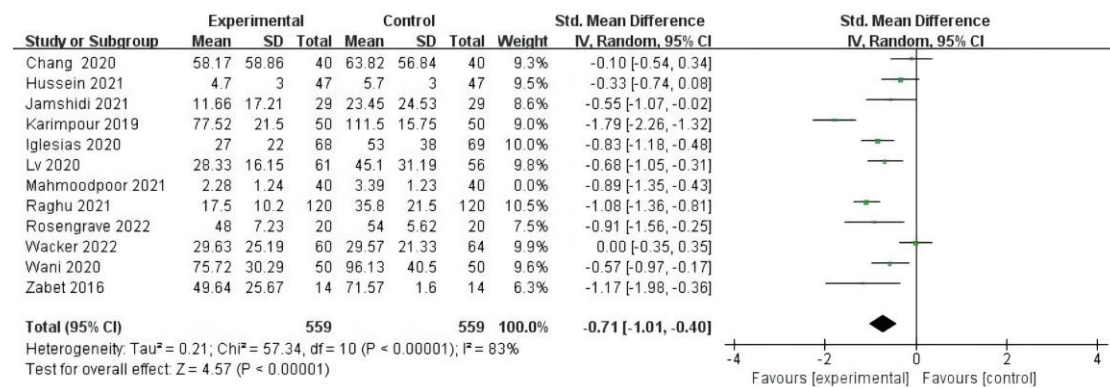

G

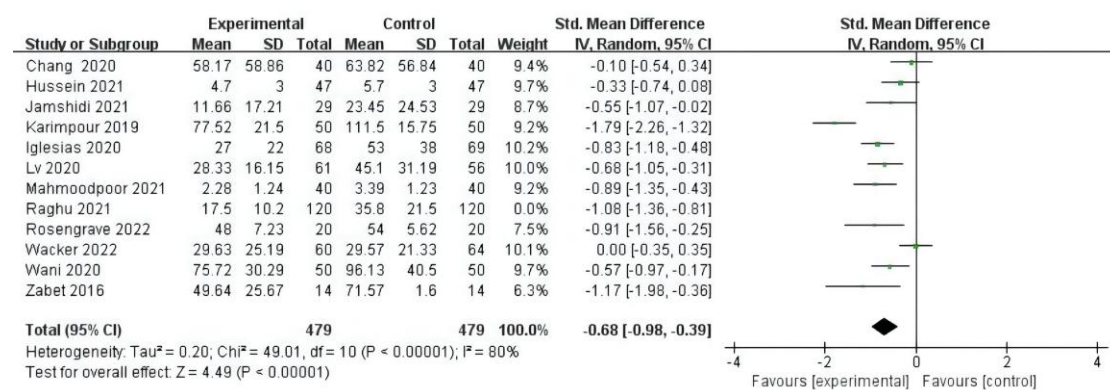

H

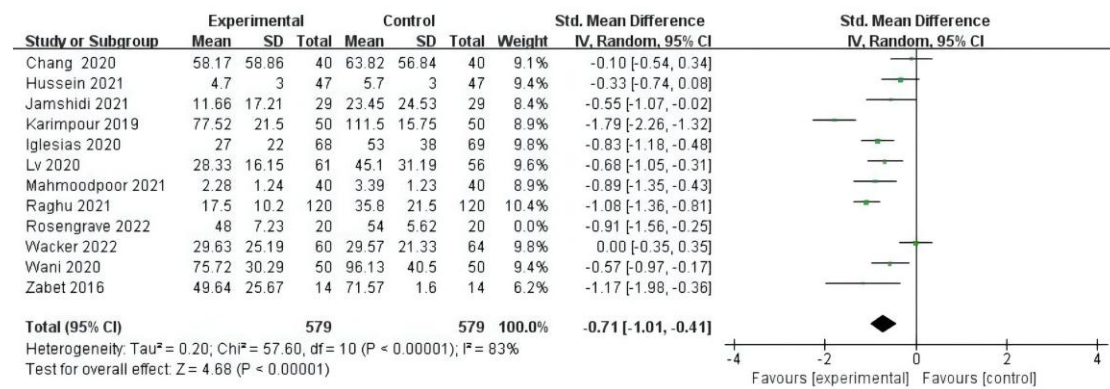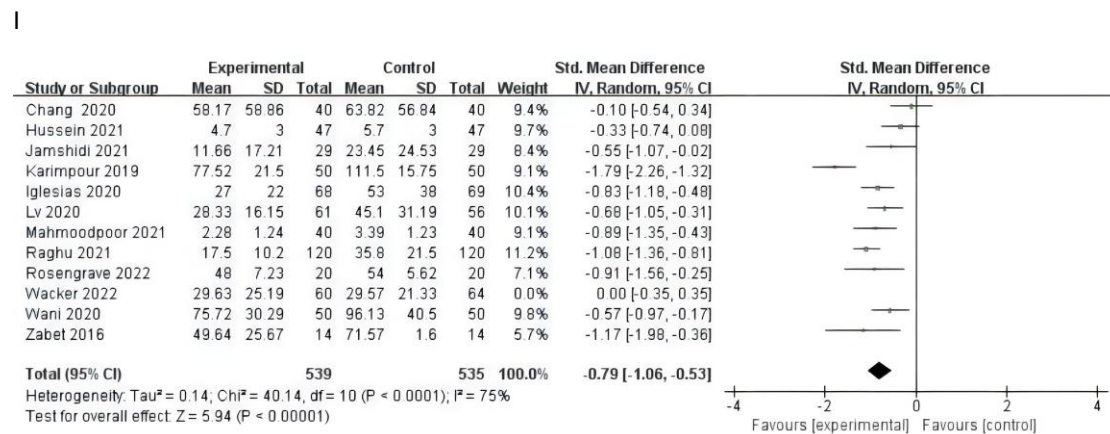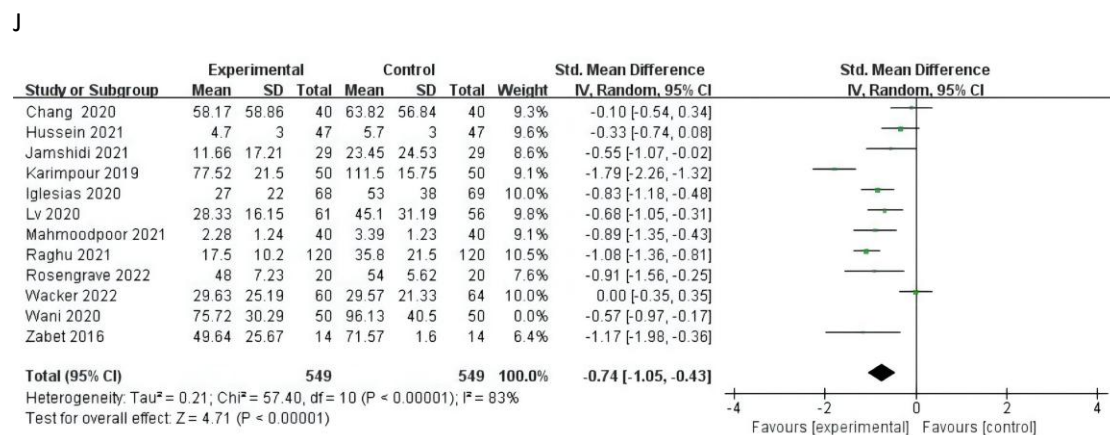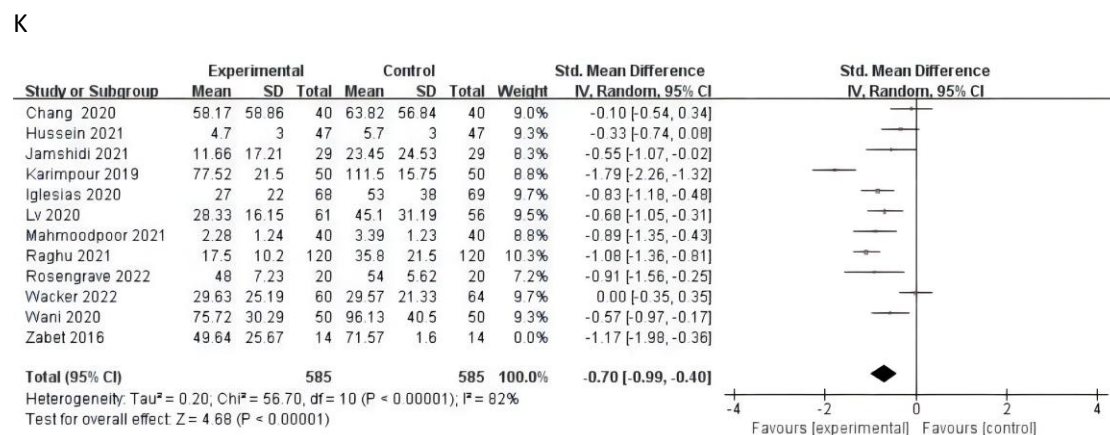

Supplementary figure 7. Sensitivity analysis for vasopressors duration

Supplement: Supplementary Materials — Supplementary Figure 1: Forest plots for the vitamin C effect on the overall mortality after removing the study of Raghu et al. Supplementary Figure 2: Forest plots for subgroup analysis of overall mortality according to protocol (A), the quality of the studies (B), blind method (C), and publication year (D). Supplementary Figure 3: Funnel plot for publication bias, which compared the 28-day mortality in the vitamin C group and control group. Supplementary Figure 4: Funnel plot for publication bias, which compared the hospital mortality in the vitamin C group and control group. Supplementary Figure 5: Forest plots for the vitamin C effect on the hospital mortality after removing the study of Raghu et al. Supplementary Figure 6: Funnel plot for publication bias, which compared the vasopressors duration in the vitamin C group and control group. Supplementary Figure 7: Sensitivity analysis for vasopressors duration. Supplementary Figure 8: Sensitivity analysis for duration of mechanical ventilation. After removal of the study of Mahmoodpoor et al. [17], the heterogeneity of the included studies significantly decreased, and the result was also changed (E). Supplementary Figure 9: Funnel plot for publication bias, which compared the length of ICU stay in the vitamin C group and control group. Supplementary Figure 10: Funnel plot for publication bias which compared the hospital stay in the vitamin C group and control group. Supplementary Figure 11: Sensitivity analysis for length of hospital stay. After removal of the study of Raghu et al., the heterogeneity changed significantly, but the result did not change (F). Supplementary Figure 12: Forest plots for the vitamin C effect on the overall mortality after removing the study of Jamshidi et al., Raghu et al., and Zabet et al. [file 6733465.f1.zip › supplementary figure 7.pdf]
